# De‐nitrosylation Coordinates Appressorium Function for Infection of the Rice Blast Fungus

**DOI:** 10.1002/advs.202403894

**Published:** 2024-05-05

**Authors:** Hong Hu, Wenhui He, Zhiguang Qu, Xiang Dong, Zhiyong Ren, Mengyuan Qin, Hao Liu, Lu Zheng, Junbin Huang, Xiao‐Lin Chen

**Affiliations:** ^1^ National Key Laboratory of Agricultural Microbiology and Provincial Key Laboratory of Plant Pathology of Hubei Province College of Plant Science and Technology Huazhong Agricultural University Wuhan 430070 China

**Keywords:** disease control, GSNOR, nitric oxide, rice blast, S‐nitrosylation

## Abstract

As a signaling molecule, nitric oxide (NO) regulates the development and stress response in different organisms. The major biological activity of NO is protein S‐nitrosylation, whose function in fungi remains largely unclear. Here, it is found in the rice blast fungus *Magnaporthe oryzae*, de‐nitrosylation process is essential for functional appressorium formation during infection. Nitrosative stress caused by excessive accumulation of NO is harmful for fungal infection. While the S‐nitrosoglutathione reductase GSNOR‐mediated de‐nitrosylation removes excess NO toxicity during appressorium formation to promote infection. Through an indoTMT switch labeling proteomics technique, 741 S‐nitrosylation sites in 483 proteins are identified. Key appressorial proteins, such as Mgb1, MagB, Sps1, Cdc42, and septins, are activated by GSNOR through de‐nitrosylation. Removing S‐nitrosylation sites of above proteins is essential for proper protein structure and appressorial function. Therefore, GSNOR‐mediated de‐nitrosylation is an essential regulator for appressorium formation. It is also shown that breaking NO homeostasis by NO donors, NO scavengers, as well as chemical inhibitor of GSNOR, shall be effective methods for fungal disease control.

## Graphical abstract

Nitric oxide (NO) is essential for Magnaporthe oryzae development and stress response. This research reveals that GSNOR‐mediated de‐nitrosylation is vital for appressorium formation and crucial for fungal infection. Proteomic analysis identifies a broad S‐nitrosylation network, with GSNOR regulating key proteins for infection. Targeting NO homeostasis presents a novel strategy for managing fungal diseases.

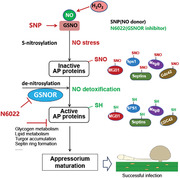

## 1 Introduction

As an intriguing signal molecule, NO controls significant biological processes, such as signal transduction, response to stress, and development.^[^ ^1^ ^]^ It can cause nitrosylation, nitrosative stress, and apoptosis through its target proteins. The balance of NO content in organisms is essential for maintaining normal cell growth.^[^ ^2^ ^]^ For example, NO acts as a key regulator of fungal morphology, pathogen, and host interaction in fungi.^[^ ^3^ ^]^ However, the detailed regulatory mechanism of NO during these processes remains largely unknown.

S‐nitrosylation is a redox‐based post‐translational modification (PTM), in which the covalent attachment of a nitric oxide (NO) moiety to a reactive cysteine thiol of a protein to form an S‐nitrosothiol (SNO).^[^ ^4^ ^]^ The degree of S‐nitrosylation of proteins in cells is largely determined by the level of S‐nitrosoglutathione (GSNO), which is the main bioactive NO donor in living organisms. GSNO acts as a stable and circulating NO reservoir, efficiently transmitting NO signals, and partially transferring it to cysteine thiol groups, leading to S‐nitrosylation.^[^ ^5^ ^]^ Emerging evidence indicates that S‐nitrosylation produces significant conformational changes in proteins, therefore plays a crucial role in regulating protein activities such as stability, biochemical activity, subcellular localization, and protein‐protein interactions.^[^ ^6^ ^]^ Thus, protein S‐nitrosylation provides a basis for physiological regulation based on changes in the redox status of cells.^[^ ^7^ ^]^

The imbalance of NO content can cause the change of S‐nitrosylation modification level of many proteins, which eventually lead to abnormal cell function. S‐nitrosoglutathione reductase (GSNOR), also known as class III alcohol dehydrogenase (ADH5) or glutathione‐dependent formaldehyde dehydrogenase (FALDH), is believed to regulate the availability of active NO in cells by converting GSNO, the reaction product of glutathione (GSH) and NO, to ammonia (NH3) and glutathione disulfide (GSSG). Thus, it can protect the body from the influence of S‐nitrosylation stress.^[^ ^8^ ^]^ The direct effect of NO on cellular pathways and the important regulation of protein S‐nitrosylation are closely related to GSNOR regulation, and an increasing number of studies have identified this enzyme as an important target for the treatment of human diseases.^[^ ^9^ ^]^

In recent years, many proteins have been identified as targets of S‐nitrosylation in various species.^[^ ^10^ ^]^ Biological functions regulated by S‐nitrosylation of some important plant proteins have also been reported, such as GAPDH, NPR1, transcription factor TGA1, and NADPH oxidase.^[^ ^11^ ^]^ In humans, S‐nitrosylation has tumor‐suppressive or tumor‐promoting effects, in particular, it is an important regulator of the tumor microenvironment (TME).^[^ ^10^, ^12^ ^]^ A large number of S‐nitrosylated modified proteins have been identified in plants using biotin switch assay, nano liquid chromatography, and tandem mass spectrometry.^[^ ^10^, ^13^ ^]^ However, the function of S‐nitrosylation and its regulatory mechanism in fungi has not been well revealed.

The filamentous fungus *Magnaporthe oryzae* is a destructive pathogen affecting the yield of cultivated rice worldwide.^[^ ^14^ ^]^ *M. oryzae* can penetrate the host epidermis by forming a special infection structure that called appressorium. Formation of functional appressorium and expansion of infectious hyphae are the key steps for *M. oryzae* to invade the host. Zhang et al. previously reported that SFA1, a GSNO reductase, regulates NO stress and formaldehyde detoxification in *M. oryzae*.^[^ ^15^ ^]^ However, the mechanism of how GSNOR removes excess NO toxicity in fungal cells remains unknown. In particular, the importance of S‐nitrosylation during infection process is unknown.

In the present study, we set out to uncovered the regulatory mechanism of S‐nitrosylation during *M. oryzae* infection. We found that NO‐triggered S‐nitrosylation played a negative role in appressorium formation, while the GSNOR‐mediated de‐nitrosylation played a positive role in this stage. Then we established a large‐scale nitrosoproteomic analysis to identify S‐nitrosylated proteins in *M. oryzae*. Some appressorium‐associated key proteins were proved to be de‐nitrosylated by GSNOR. Notably, functions of the four septins were blocked by S‐nitrosylation, which was removed by GSNOR for septin ring formation, and required for function of the appressorium. In conclusion, our findings reveal a conserved mechanism of GSNOR‐mediated de‐nitrosylation in the formation of fungal infection structure, and disrupting the NO homeostasis may be a feasible strategy for controlling diverse diseases in plants.

## 2 Results

### 2.1 Excess NO is Harmful for Functional Appressorium Formation of *M. oryzae*

**Figure 1:**
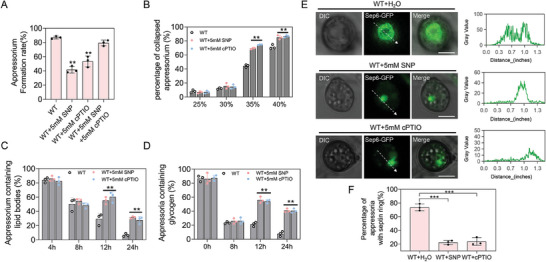
NO regulates the formation of functional appressorium. A) Appressorium formation rates of the wild‐type (WT) strains after treatment with 5 mm SNP or cPTIO. The spore suspensions treated with 5 mm SNP or cPTIO were incubated on the hydrophobic surface, and the appressarium formation rate was measured at 24 h. Data presented are the mean ± standard errors from three biological replicates (*n* = 3), and asterisks represent significant differences (** *P* < 0.01). B) Observation of appressorium turgor pressure. Conidial suspension droplets of wild‐type (WT) strains treated with SNP or cPTIO were placed on the hydrophobic surface of a coverslip and treated with different concentrations of polyethylene glycol 8000 (PEG8000) at 24 h post‐inoculation (hpi). C) Statistics of lipid percentage in conidia or appressarium during lipid development in wild‐type (WT) strains treated with SNP or cPTIO. Data presented are the mean ± standard errors from three biological replicates (*n *= 3), and significant differences compared with the WT are indicated by an asterisk (** *P* < 0.01). D) Statistics of lipid percentage in conidia or appressarium during glycogen development in wild‐type (WT) strains treated with SNP or cPTIO. Data presented are the mean ± standard errors from three biological replicates (*n* = 3), and significant differences compared with the WT are indicated by an asterisk (** *P* < 0.01). E) Observation on the formation of appressorium septin ring after treatment with 5 mm SNP or cPTIO. Bar, 5 µm. F) Percentage of septin‐ring formation in wild‐type (WT) strains treated with 5 mm SNP or cPTIO. Data presented are the mean ± standard errors from three biological replicates (*n* = 3), and significant differences compared with the WT are indicated by an asterisk (*** *P* < 0.001).

To explore the possible role of NO in *M. oryzae*, we detected the effect of NO on development and infection process of the wild‐type strain P131. Sodium nitroprusside (SNP) is an effective NO donor that releases NO into the cell.^[^ ^16^ ^]^ We treated the conidia suspensions of *M. oryzae* on hydrophobic surface with 5 mM SNP, which resulted in a significant reduction of appressorium formation ratio (**Figure** **1**A). SNP‐treatment also led to form immature appressoria, demonstrated by reduced turgor formation (Figure 1B), lipid and glycogen utilization (Figure 1C,D; Figure S1A,B, Supporting Information), and abnormal septin ring formation (Figure 1E,F). Carboxy PTIO (cPTIO, 2‐(4‐carboxyphenyl)−4,5‐dihydro‐4,4,5,5‐tetramethyl‐1H‐imidazolyl‐1‐oxy‐3‐oxide) is a commonly used NO scavenger.^[^ ^17^ ^]^ When we used 5 mm cPTIO to treat the conidia suspension containing 5 mm SNP on hydrophobic surface, the NO level was successfully reduced, and the appressorium formation and maturation was comparable to the wild type. However, 5 mm cPTIO alone treatment also resulted in reduction of functional appressorium formation (Figure 1C,D; Figure S1A,B, Supporting Information). These data suggested that high concentration of NO acts as a negative regulator for appressorium formation and maturation.

### 2.2 Identification of an S‐nitrosoglutathione Reductase GSNOR in *M. oryzae*

**Figure 2:**
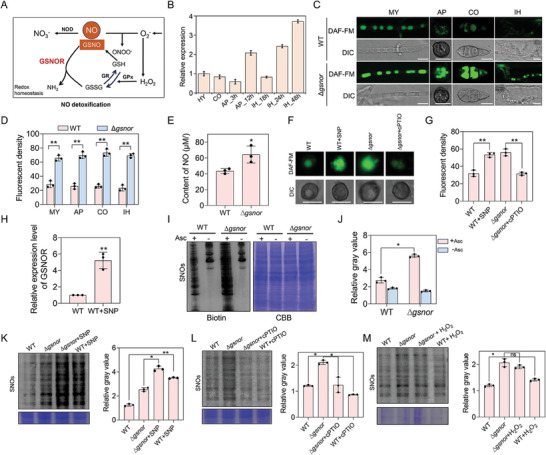
NO and GSNOR conversely regulates S‐nitrosylation of *M. oryzae*. A) Cellular NO detoxification pathway. B) Expression of *GSNOR* gene in different developmental and infection stages of *M.oryzae*. HY: Mycelial hyphae; CO: Conidia; AP_3h: appressoria at 3 hpi (hours post inoculation); AP_12h: appressoria at 12 hpi; IH_18h: invasive hyphae at 18 hpi; IH_24h: invasive hyphae at 24 hpi; IH_48h: invasive hyphae at 48 hpi. Error bars represent standard errors. C) Observation of NO content in wild‐type (WT) and Δ*gsnor* at different developmental stages after DAF‐FM DA staining. Bar,10 µm. D) The bar chart shows the fluorescence intensity of DA‐FM DA staining at different developmental stages. Samples of each strain were measured using the ImageJ software. Data presented are the mean ± standard errors from three biological replicates (*n* = 3), and significant differences compared with the WT are indicated by an asterisk (** *P* < 0.01). E) The concentration of NO in the mycelia of wild‐type (WT) and Δ*gsnor* was determined by the improved Griess method. Data presented are the mean ± standard errors from three biological replicates (*n* = 3), and significant differences compared with the WT are indicated by an asterisk (* *P* < 0.05). F) Observation of NO content in wild‐type (WT) and Δ*gsnor* during appressorium after SNP and cPTIO treatment. Bar,10 µm. G) The bar chart shows the fluorescence intensity of DA‐FM DA staining at appressoria stage. Samples of each strain were measured using the ImageJ software. Data presented are the mean ± standard errors from three biological replicates (*n* = 3), and significant differences compared with the treatment are indicated by an asterisk (** *P* < 0.01). H) Expression of the *GSNOR* gene after SNP treatment in the wild‐type, and the ACTIN gene was used as an internal control. Data presented are the mean ± standard errors from three biological replicates (*n* = 3), and significant differences compared with the SNP treatment are indicated by an asterisk (** *P* < 0.01). I) Total levels of S‐nitrosylated proteins in wild‐type (WT) and Δ*gsnor*. Hypha at 7 days of age were used as samples for analysis. Asc, Sodium ascorbate; SNOs, S‐nitrosylated proteins. J) Band quantification of the immunoblot showing a specific increase of S‐nitrosylation for wild‐type (WT) and Δ*gsnor* proteins. K) Total levels of S‐nitrosylated proteins in wild‐type (WT) and Δ*gsnor* after SNP treatment. Hypha at 7 days of age were used as samples for analysis. L) Total levels of S‐nitrosylated proteins in wild‐type (WT) and Δ*gsnor* after cPTIO treatment. Hypha at 7 days of age were used as samples for analysis. M) Total levels of S‐nitrosylated proteins in wild‐type (WT) and Δ*gsnor* after H_2_O_2_ treatment. Hypha at 7 days of age were used as samples for analysis. Data presented are the mean ± standard errors from three biological replicates (*n* = 3), and significant differences compared with no treatment are indicated by an asterisk (** *P* < 0.01). ns, not significant.

In order to infect the host successfully, *M. oryzae* must developed NO detoxification mechanism for appressorium formation. An S‐nitrosoglutathione (GSNO) reductase (GSNOR) converts GSNO to ammonia (NH3) and glutathione disulfide (GSSG) is commonly used for nitrosative stress detoxification in eukaryotes (**Figure** **2**A). It has been revealed that the *GSNOR* encodes a S‐nitrosoglutathione reductase involved in the regulation of S‐nitrosylation in Arabidopsis and humans.^[^ ^18^ ^]^ To investigate appressorial NO detoxification mechanism in *M. oryzae*, we identified the homologous protein of GSNOR in *M. oryzae* (MGG_06011), which contains an alcohol dehydrogenase domain (ADH) at the N terminus and another ADH domain with a zinc finger at the C terminus (Figure S2A, Supporting Information). GSNOR is highly conserved in various pathogenic fungi, as well as in bacteria *Escherichia coli*, *Saccharomyces cerevisiae*, *A. thaliana*, *Oryza sativa*, *Drosophila melanogaster*, mammals and humans (Figure S2B, Supporting Information). We successfully disrupted the *M. oryzae GSNOR* gene in the wild‐type strain P131 through a split‐PCR method (Figure S3A, Supporting Information). The transformants were screened by PCR‐mediated method, and RT‐PCR results showed that two mutants (KO1 and KO2) were lack of *GSNOR* (Figure S3B, Supporting Information). The complementary transformants were also successfully obtained by introducing *GSNOR* driven by its native promoter into the Δ*gsnor* mutant.

We detected expression profile of *GSNOR* in different developmental stages and infection processes. *GSNOR* was stably expressed at all stages, with a significant increase in AP_12 h, and the highest expression at IH_24 h and IH_48 h (Figure 2B). These data suggest that *GSNOR* could play more important roles in appressorium and infectious hyphae of *M. oryzae*. GFP‐GSNOR and the mitochondrial marker Mito‐tracker partially co‐localize in the mycelium and conidia (Figure S4, Supporting Information), indicating that GSNOR mainly localizes in the mitochondria and cytoplasm, consistent with its predicted function as a class III alcohol dehydrogenase.^[^ ^19^ ^]^

### 2.3 GSNOR is Required for Equilibrium of NO in *M. oryzae*

To explore the possible role of GSNOR in NO detoxification process, we observed NO level of the wild‐type strain and Δ*gsnor* mutant by DAF‐FM (4‐amino‐5‐methylamino‐2,7‐difluorofluorescein diacetate) staining assay at different developmental stages. DAF‐FM has been reported as an established, specific probe for the detection of intracellular NO.^[^ ^3^, ^20^ ^]^ The results showed that, compared with that of the wild‐type strain, NO levels of the Δ*gsnor* mutant in all tested samples, including mycelium, conidium, appressorium, and invasive hypha, were significantly increased (Figure 2C,D). The NO content in the mycelium was also determined using the improved Griess method, and it was found that the NO content in Δ*gsnor* mutant significantly increased (Figure 2E). This suggests the presence of NO at various developmental stages of *M. oryzae*, indicating the importance of this signaling molecule in the life cycle of *M. oryzae*. The NO level of the Δ*gsnor* mutant was reduced by addition of 1 mm NO scavenger cPTIO (Figure 2F,G). To investigate whether *GSNOR* responds to NO level, we examined the expression level of *GSNOR* after SNP treatment. The qRT‐PCR results indicate that NO can induce the expression of *GSNOR* in *M. oryzae* (Figure 2H). These results showed that deletion of *GSNOR* resulted in a significant increase of NO level in *M. oryzae*, suggesting that GSNOR is required for detoxification of intracellular NO to keep its balance.

### 2.4 NO and GSNOR Conversely Regulates S‐nitrosylation

In cells, NO and its main bioactive donor S‐nitrosoglutathione (GSNO) can transduce NO signals to a cysteine thiol through S‐nitrosylation,^[^ ^21^ ^]^ while GSNOR proteins are denitrosylases catalyzing de‐nitrosylation process and converts GSNO into ammonia and oxidized glutathione (GSSG) (Figure 2a).^[^ ^22^ ^]^ We hypothesized that the increase of NO level in *M. oryzae* should lead to a greater degree of S‐nitrosylation of proteins. To test this hypothesis, the biotin‐switch assay was used in this study to verify the difference in total S‐nitrosylation levels. Total proteins of mycelia were extracted for the biotin‐switch assay, the biotinylated proteins were then immunoblotted with an anti‐biotin antibody to detect the S‐nitrosoylation level. The result showed that total S‐nitrosylation level in Δ*gsnor* was much higher than that in WT (Figure 2I,J), suggesting that *GSNOR* was involved in de‐nitrosylation of *M. oryzae*.

Since SNP and cPTIO can respectively increase and decrease the content of NO in the cells of *M. oryzae*, we suspect that SNP and cPTIO can also affect the S‐nitrosoylation level of *M. oryzae*. We treated the hyphae of wild‐type and Δ*gsnor* with 1 mm SNP, 1 mm cPTIO, and 10 mm H_2_O_2_. The results showed that total S‐nitrosylation levels of WT with 1 mm SNP were significantly increased, but evidently decreased in which treated with 1 mm cPTIO (Figure 2K,L). S‐nitrosylation levels of Δ*gsnor* treated with 1 mm SNP were also significantly increased, but evidently decreased in which treated with 1 mm cPTIO (Figure 2K,L). Therefore, we conclude that NO and GSNOR positively and negatively regulate S‐nitrosylation modification, respectively.

### 2.5 GSNOR‐Mediated NO Balance is Required for Maintaining Redox Homeostasis in *M. oryzae*

H_2_O_2_ and NO interact with each other as endogenous signaling molecules in plants and animals. In order to elucidate the relationship between H_2_O_2_ and NO in *M. oryzae*, we treated the mycelia of WT with 10 mm H_2_O_2_, and stained with DAF FM to detect the NO level. We found that treatment of H_2_O_2_ caused an NO burst in wild‐type cells (Figure S5A–C, Supporting Information), suggesting that H_2_O_2_ stimulates NO production in *M. oryzae*. The degree of S‐nitrosylation of WT was also slightly enhanced in H_2_O_2_ condition (Figure 2M).

Since GSNOR‐mediated S‐nitrosylation is a redox modification, GSNOR may be involved in maintaining redox homeostasis of *M. oryzae*. Glutathione is either reduced glutathione (GSH) or oxidized glutathione disulfide (GSSG) forms. GSSG is reduced to GSH by glutathione reductase. Reduced glutathione plays an important role in maintaining the proper redox state of sulfhydryl groups in proteins and is a key antioxidant. Our data showed that the content of GSH in ∆*gsnor* mutant was significantly lower than that of the wild type, and the ratio of GSH/GSSG was also significantly decreased (Figure S5D,E, Supporting Information). Moreover, exogenous GSH did not impact the level or content of NO in *M. oryzae* (Figure S5F,G,H, Supporting Information), likely due to the fact that GSH is an inactive NO donor. These results indicate the importance of GSNOR‐mediated NO balance in regulating the redox status of *M. oryzae*.

### 2.6 GSNOR is a Positive Regulator of Virulence

**Figure 3:**
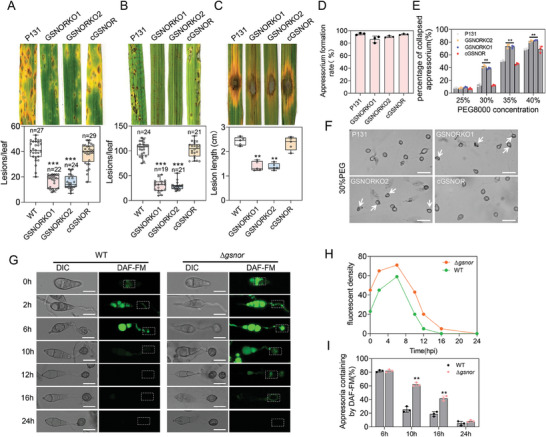
GSNOR is responsible for full virulence. A) Lesions formed by wild‐type (WT) and Δ*gsnor* on barley leaves at 5 days (dpi) after inoculation. The lesion number was examined at 5 days (dpi) after inoculation. B) Lesions formed by wild‐type (WT) and Δ*gsnor* on rice seedlings at 5 days (dpi) after inoculation. The lesion number was examined at 5 days (dpi) after inoculation. C) Lesions formed by wild‐type (WT) and Δ*gsnor* on wounded rice leaves. Hyphal agar plugs (5 mm diameter) were placed on rice leaves treated with wounds and incubated for 4 days (dpi). The lesion length was examined at 4 days (dpi) after inoculation. D) Statistical analysis of appressorium formation rate in 24 h period. Data presented are the mean ± standard errors from three biological replicates (*n* = 3). E) Cytorrhysis assay for appressorium turgor pressure. Data presented are the mean ± standard errors from three biological replicates (*n* = 3), and asterisks represent significant differences (** *P *< 0.01). F) Observation of appressorium turgor pressure. Conidial suspension droplets were placed on the hydrophobic surface of a coverslip and treated with different concentrations of polyethylene glycol 8000 (PEG8000) at 24 hpi. G) The levels of NO during conidial to appressorial formation were detected by DAF‐FM DA. The conidia were stained after inoculation on hydrophobic cover slips for 2, 6, 10, 12, 16, and 24 h. H) The fluorescence intensity of NO levels at the indicated regions during conidial to appressorial formation in wild‐type and Δ*gsnor* strains was measured using DAF‐FM DA staining at different time points. I) Statistics of the percentage of wild‐type (WT) and Δ*gsnor* strains stained by DAF‐FM DA in conidia or appressorial during NO metabolism. Data in (A–C) are displayed as box and whisker plots with individual data points: center line, median; box limits, and asterisks represent significant differences (** *P *< 0.01, *** *P *< 0.001).

Subsequently, we detected the effect of *GSNOR* deletion on the virulence of *M. oryzae*. Compared with WT and the complementary strains, the virulence of ∆*gsnor* was significantly reduced on barley and rice (**Figure** **3**A,B), and the lesions caused by ∆*gsnor* to rice wounds were significantly smaller (Figure 3C). In conclusion, these results demonstrate that *GSNOR* is important for the infection and full virulence of *M. oryzae*.

Since cPTIO treatment caused a decrease in the S‐nitrosylation level of Δ*gsnor*, we hypothesized that cPTIO might rescue the defects of Δ*gsnor*. Inoculation of Δ*gsnor* after cPTIO treatment showed that cPTIO could partially restore the infection ability of Δ*gsnor* (Figure S6A,B,C, Supporting Information). At the same time, we observed that cPTIO reduced the NO level in the infectious hyphae of Δ*gsnor* (Figure S6D,E, Supporting Information), and therefore, this may be caused by the removal of excess NO content in Δ*gsnor* by cPTIO. These results suggest that cPTIO was able to partially restore the infection defect of Δ*gsnor*.

### 2.7 GSNOR Plays an Important Role in Appressorium Maturation

Considering that deletion of *GSNOR* significantly resulted in accumulation of appressorial NO, we wonder if GSNOR is important for functional appressorium formation. There was no significant difference in the formation of appressorium between WT and ∆*gsnor* (Figure 3D), but appressorium maturation of the ∆*gsnor* mutants was significantly affected. The turgor pressure was tested by using PEG8000‐treatment showed that the appressorium of ∆*gsnor* mutants was much easier to collapse than that of WT (Figure 3E,F), indicating that accumulation of turgor pressure in appressorium of the mutant was evidently reduced.

Glycogen and lipid stores in conidia are essential nutrients for the appressorium maturation of *M. oryzae*. Since ∆*gsnor* is defective in accumulation of turgor, we speculate that glycogen and lipid utilization may be blocked in ∆*gsnor* mutants. Therefore, I_2_/KI and Nile Red staining were used to observe the distribution of glycogen and lipid in cells at different time points. The results showed that after 18 hpi, the glycogen and lipid could be still detected in the ∆*gsnor* mutant but not in WT (Figure S7A–D, Supporting Information), indicating that blocking NO metabolism also affects glycogen and lipid metabolic processes. Taken together, *GSNOR* plays an important role in appressorium maturation of *M. oryzae*.

### 2.8 GSNOR‐Mediated de‐nitrosylation is Essential for NO Detoxification During the Development of Appressorial in *M. oryzae*

Since a suitable dose of NO is required during appressorial formation in *M. oryzae*, high doses of NO are detrimental to *M. oryzae* (Figure 1A–D). To better understand the function of GSNOR in NO detoxification in pathogen, we investigated the dynamics of NO during appressorial development in *M. oryzae*. We found that NO levels reached a maximum during appressorial germination in the wild‐type and that NO was gradually and completely metabolised during subsequent development and maturation (Figure 3G–I). Notably, this dynamic is similar to the ROS metabolism process of appressorial in the wild type *M. oryzae*, suggesting that RNS metabolism is also present in fungal cells. Green fluorescence was more intense in the germ tubes and early appressorial formation in the ∆*gsnor* mutant than in the wild‐type, while NO was still detectable in the ∆*gsnor* mutant at later stages of appressorial development (Figure 3–I). These results suggest that GSNOR is a key regulator of NO detoxification during appressorial development and pathogenesis of *M. oryzae*.

### 2.9 GSNOR is Required for Oxidative Stress Detoxification

We investigated whether GSNOR is required for response to various environmental stresses. The results showed that ∆*gsnor* was not sensitive to cell wall inhibitors of (0.1 mg ml^−1^ Calcofluor White [CFW], 0.2 mg ml^−1^ Congo Red [CR]), and osmotic stresses (0.5 m NaCl, 1.0 m Sorbitol). Notably, we found that the ∆*gsnor* mutant was more sensitive to oxidative stress (10 mm H_2_O_2_) (Figure S8A,B, Supporting Information), with a concentration dependent (Figure S8C,D, Supporting Information). These results suggested that *GSNOR* is required for oxidative stress detoxification.

### 2.10 Identification of S‐nitrosylated Proteins by indoTMT Switch Labeling

**Figure 4:**
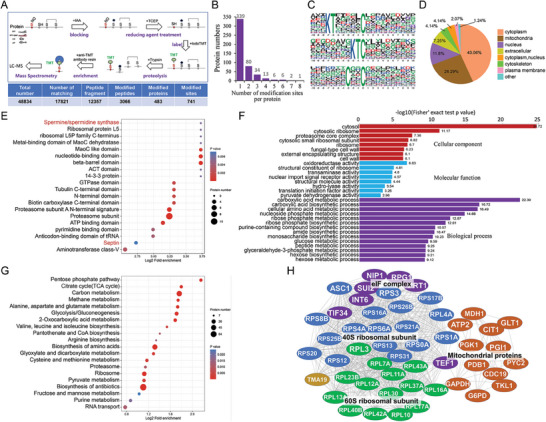
Identification of S‐nitrosylated proteins by indoTMT switch labeling. A) Schematic illustration of S‐nitrosoproteomic analysis procedure and its parameter statistics. For detailed notes on these proteins, see Table S1 (Supporting Information). B) Statistics of the number of individual protein modification sites discovered by S‐nitrosoproteomic analysis. C) Presumptive consensus sequences of the S‐nitrosylation motifs derived from the analysis of 741 S‐nitrososylated protein sites. D) Localization and classification of S‐nitrosylation target proteins. E) Enrichment of S‐nitrosylation target protein domains. F) GO analysis of significantly altered S‐nitrosylated proteins by cellular component, molecular function and biological process. G) Enrichment analysis of S‐nitrosylated proteins KEGG signaling pathway. H) Protein‐protein interaction network of the S‐nitrosylated proteins.

In order to reveal the regulatory mechanism of S‐nitrosylation in pathogenesis through the target proteins, we performed a proteome analysis to identify S‐nitrosylation modified proteins. We used an iodoTMT labeling method combining with tandem mass spectrometry to characterize the S‐nitrosylated proteins in *M. oryzae*. A mixed samples containing mycelia, conidia, appressoria and invasive hyphae, as well as 1 mM SNP‐treated mycelia was used for total protein extraction (**Figure** **4**A). In two independent experiments, we identified 741 S‐nitrosylation modified sites in 483 proteins (Figure 4A; and Table S1, Supporting Information). Among these proteins, 339 contain 1 site, 80 contain 2 sites, and 62 contain 3–8 sites (Figure 4B). Next, we sought to identify consensus motifs for Cys sites. The main S‐nitrosylation motifs identified in this work with high frequency were xxxKTxxxxxCxxxxxxxxxx, xxxxxxxxKTCxxxxxxxxxx, xxxxxxxCxxCxxxxxxxxxx (Figure 4C). Subcellular localization annotation of the S‐nitrosylated proteins showed that they were mostly located in cytoplasm (43.06%), mitochondria proteins (26.29%), and nucleus proteins (11.8%) were also commonly identified as S‐nitrosylated proteins (Figure 4D).

### 2.11 Functional Classification of the S‐nitrosylated Proteins

Functional structural domain enrichment analysis showed that spermine/spermidine synthase, ribosomal proteins, nucleotide‐binding domain, GTPase domain, proteasomal subunit domain, septin domain, pyrimidine binding domain, anticodon‐binding of tRNA domain, and aminotransferase class‐v domain, etc. were enriched (Figure 4E). Interestingly, proteins containing spermine/spermidine synthase (Sps1) and septin domains (septin proteins) have been found to be important for appressorium function in *M. oryzae*.^[^ ^23^ ^]^

Gene ontology (GO) analysis revealed S‐nitrosylated target proteins were involved in numerous biological processes, mainly involving the carboxylic acid biosynthetic and metabolic process, cellular amino acid metabolic process, nucleoside phosphate metabolic process, ribose phosphate biosynthetic and metabolic process, amide biosynthetic process, hexose biosynthetic and metabolic process, glucose metabolic process, peptide metabolic process, glyceraldehyde‐3‐phosphate metabolic process, etc. (Figure 4F). For cellular components, the highest proportions of cytosol, cytosolic ribosome, proteasome core complex, and cell wall were found. For molecular functions, oxidoreductase activity, structural constituent of ribosome, transaminase activity, nuclear import signal receptor activity, and hydro‐lyase activity were enriched (Figure 4F).

The KEGG enrichment analysis showed that some mitochondria‐related pathways were enriched, including pentose phosphate pathway, citrate cycle, methane metabolism, amino acid biosynthesis and metabolism, pantothenate and CoA biosynthesis, glyoxylate and dicarboxylate metabolism, pyruvate metabolism, etc. (Figure 4G). This S‐nitrosylation pattern suggested that S‐nitrosylation plays an important role in cell biosynthesis and energy metabolism in *M. oryzae*.

### 2.12 Analysis of S‐nitrosylation‐Mediated Protein‐Protein Interaction Network

Protein‐protein interaction (PPI) network analysis showed that a large number of S‐nitrosylation target proteins interacted and clustered into proteasome components, ribosome components, tRNA synthetases, key enzymes of the tricarboxylic acid cycle and lipid metabolism‐related enzymes (Figure 4H), These data suggested that S‐nitrosylated may play key roles in the proteasome system, ribosome system and mitochondrial system through simultaneously modifying key components.

### 2.13 Pathogenesis‐Related Appressorium Proteins are Targets of S‐nitrosylation

**Figure 5:**
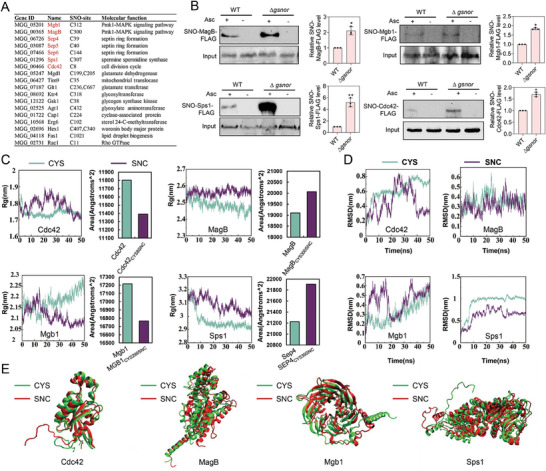
S‐nitrosylation regulates proteins related to appressorium function. A) S‐nitrosylated proteins associated with the pathogenesis of rice blast fungus. B) Accumulation of S‐nitrosylated MAGB‐FLAG, MGB1‐FLAG, SPS1‐FLAG and CDC42‐FLAG in wild‐type (WT) and Δ*gsnor*. The blots were detected by anti‐FLAG antibody. Input protein levels were also determined using anti‐FLAG antibody, respectively. Asc, sodium ascorbate; SNO, S‐nitrosylated proteins. A quantitative analysis of the data is shown at the right. Data presented are the mean ± standard errors from three biological replicates (*n* = 3), and significant differences compared with the WT are indicated by an asterisk (* *P* < 0.05; ** *P* < 0.01). C) Analysis of protein radius of gyration (Rg, left) and solvent‐accessible surface area (right) of proteins during molecular dynamics simulations. D) Analysis of root mean square deviation (RMSD) of protein backbone during molecular dynamics simulations. E) Structural alignment of proteins with or without S‐nitrosylation by dynamics simulations.

Our S‐nitrosoproteomic analysis revealed that many proteins related to the pathogenesis of *M. oryzae* were identified as S‐nitroitylation target proteins (**Figure** **5**A; and Table S2, Supporting Information). Interestingly, some of them were key pathogenesis‐related proteins involved in appressorium formation and maturation, including proteins of Pmk1‐MAPK signaling pathway (Mgb1/C312, MagB/C300, Cdc42/C8), glycogen and lipid metabolism (Gsk1/C38, Fas1/C1021), spermine spermidine synthase (Sps1/C307), woronin body protein (Hex1/C340 and C407), glyoxylate aminotransferase (Agt1/C432), cyclase‐associated protein (Cap1/C224), and sterol 24‐C‐methyltransferase (Erg6/C102). Four reported key appressorium‐associated proteins (Mgb1, MagB, Cdc42, and Sps1) were selected for confirmation of S‐nitrosylation by a biotin‐switch assay. All these four proteins were indeed S‐nitrosylated in both wild‐type strain and ∆*gsnor* mutant, and the accumulation of S‐nitrosylation of these four proteins in ∆*gsnor* mutant was significantly increased compared to the wild‐type (Figure 5B). These results suggested that key proteins involved in appressorium formation are S‐nitrosylated proteins in *M. oryzae*.

To gain a deeper understanding of the potential mechanisms by which S‐nitrosylation could impact the structural characteristics of its target proteins, proteins of Mgb1, MagB, Sps1, and Cdc42 were used for molecular dynamics simulations. S‐nitrosylation of the cysteine (CYS) residues at the modification sites of target proteins resulted in S‐nitroso‐cysteine (SNC). In general, the Root Mean Square Deviation (RMSD) ≤0.3 nm during a 20 ns MD run indicates strong complex stability.^[^ ^24^ ^]^ Radius of gyration (Rg) value was used to describe the structural integrity and folding behavior of the proteins.^[^ ^25^ ^]^ We found that the conformations of the S‐nitrosylation target proteins have evidently changed. Cdc42_CYS8SNC_ exhibited a lower Rg value compared to Cdc42, indicating that the S‐nitrosylated Cdc42 protein adopts a more compact structure, resulting in a reduced solvent‐accessible surface area and an increase in the hydrophobic area (Figure 5C). The RMSD analysis of the target proteins during molecular dynamics simulation process showed that the simulation had basically reached equilibrium (Figure 5D). Similar result was also found in Mgb1. While, the S‐nitrosylated MagB and Sps1 exhibited a higher Rg value compared to their non‐nitrosylated forms, adopted much looser structures, resulting in an increased solvent‐accessible surface area and an increase in the hydrophilic area. This alteration may affect the interactions between the target protein and substrate.

### 2.14 Functions of Septin Proteins are Negatively Regulated by S‐nitrosylation

**Figure 6:**
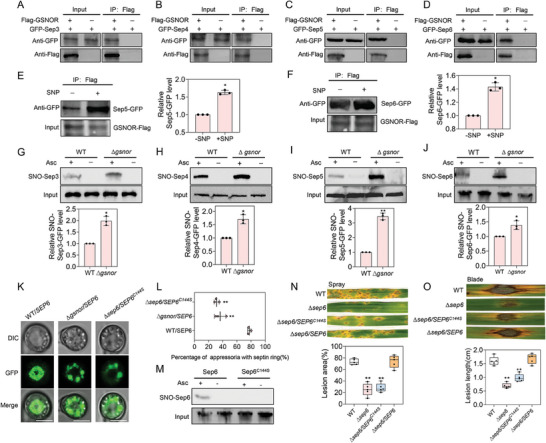
S‐nitrosylation regulates functions of septins in *M. oryzae*. A–D) Co‐immunoprecipitation analyses between GSNOR and Sep3 (A), Sep4 (B), Sep5 (C), and Sep6 (D). In proteins eluted from anti‐FLAG beads, GFP‐Sep3, GFP‐Sep4, GFP‐Sep5, and GFP‐Sep6 bands were also detected by anti‐ GFP antibody in the transformant expressing the FLAG construct. E,F) Co‐immunoprecipitation analyses of GSNOR with Sep5 (E) and Sep6 (F). Proteins were extracted from the wild‐type strains containing Sep5‐GFP, Sep6‐GFP, and GSNOR‐3×FLAG constructs in liquid CM with or without SNP treatment, and were immunoprecipitated with anti‐FLAG beads. Input protein levels were also determined using anti‐FLAG antibody. The bands of GFP‐Sep5 and GFP‐Sep6 with or without SNP treatment were detected by anti‐GFP antibody. G–J) Accumulation of S‐nitrosylated Sep3‐GFP (G), Sep4‐GFP (H), Sep5‐GFP (I), and Sep6‐GFP (J) in WT and Δ*gsnor*. The blots were detected by anti‐GFP antibody. Input protein levels were also determined using anti‐GFP antibody, respectively. Asc, sodium ascorbate; SNO, S‐nitrosylated proteins. A quantitative analysis of the data is shown below the blot. Data presented are the mean ± standard errors from three biological replicates (*n* = 3), and significant differences compared with the WT are indicated by an asterisk (* *P* < 0.05; ** *P* < 0.01). K) Localization of Sep6‐GFP and Sep6^C144S^‐GFP in appressorium. Bar, 5 µm. L) Percentage of septin‐ring formation in wild‐type (WT), *∆gsnor /SEP6*, and *∆sep6/SEP6^C144S^* strains. Data presented are the mean ± standard errors from three biological replicates (*n* = 3), and significant differences compared with the WT are indicated by an asterisk (** *P* < 0.01). M) S‐nitrosylation verification of Sep6‐GFP and Sep6^C144S‐GFP^ proteins in vivo. N) Diseased spots formed by different strains on barley leaves. Spore solution was sprayed on barley plants and cultured for 5 days to observe the disease spots. O) Lesions formed on scratched rice leaves by different strains. Hyphal agar plugs were placed onto rice leaves with treatment of wounds and incubated for 5 d. Data in (N‐O) are displayed as box and whisker plots with individual data points: center line, median; box limits, and asterisks represent significant differences (** *P *< 0.01).

Previous studies have shown that the normal formation of septin rings in the appressorium of *M. oryzae* is crucial for plant penetration and intracellular cell‐to‐cell movement.^[^ ^23^, ^26^ ^]^ Notably, three septin proteins have also been identified as S‐nitrosylated proteins (Sep4 at C39, Sep5 at C40, and Sep6 at C144) according to our S‐nitrosoproteomic analysis (Figure 5A). We speculate that Sep3 may be also regulated by S‐nitrosylation. Co‐immunoprecipitation and yeast two hybrid experiments both showed that all the septins could interact with GSNOR (**Figure** **6**A–D; Figure S9, Supporting Information) and that the interactions of GSNOR with septin proteins in vivo were enhanced by SNP treatment (Figure 6E,F). These results indicate that S‐nitrosylation modifications modulate protein interactions in *M. oryzae*. The biotin‐switch assay showed that all four septin proteins were S‐nitrosylated and the degree of modification in ∆*gsnor* mutant were all significantly higher than that of the wild‐type strain (Figure 6G–J), suggesting that GSNOR could induce de‐nitrosylation of septin proteins.

In order to explore the possible biological significance of S‐nitrosylation in septin proteins, as an example, we analyzed the subcellular localization of Sep6 protein in the wild‐type strain and ∆*gsnor* mutant. We observed that in the wild‐type strain, the GFP signal accumulated in the center of appresorium as a ring, while in the ∆*gsnor* mutant, GFP‐Sep6 was distributed throughout the appresorium mainly in the form of granules without forming a ring (Figure 6K,L). Overall, we confirmed that septin proteins is the target of S‐nitrosylation, and that whose function can be regulated by S‐nitrosylation through affecting their subcellular localization.

### 2.15 Structural Analysis of S‐nitrosylation Sites in Septin Proteins by Molecular Dynamics Simulation

**Figure 7:**
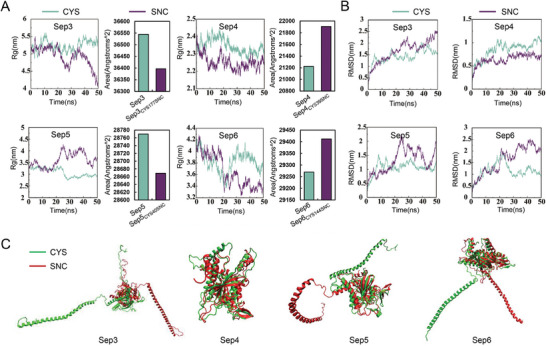
Structural modeling of septin proteins. A) Analysis of protein radius of gyration (Rg, left) and solvent‐accessible surface area (right) of proteins during molecular dynamics simulations. B) Analysis of root mean square deviation (RMSD) of protein backbone during molecular dynamics simulations. C) Structural alignment of proteins with or without S‐nitrosylation by dynamics simulations.

To gain a deeper understanding of the potential mechanisms by which S‐nitrosylation could impact the structural characteristics of septins, we also conducted molecular dynamics simulations. We found that Sep5_CYS40SNC_ exhibited a higher Rg value compared to its non‐nitrosylated forms, indicating that the Sep5_CYS40SNC_ adopts a much looser structure, resulting in a decreased in solvent‐accessible surface area and an increase in the hydrophobic area (**Figure** **7**A–C). Conversely, Sep3_CYS177SNC_,Sep4_CYS39SNC_, and Sep6_CYS144SNC_ exhibited a lower Rg value compared to theirs non‐nitrosylated forms, indicating these septins adopts a more compact structure. Resulting in a decreased solvent‐accessible surface area and an increase in the hydrophobic area of Sep3_CYS177SNC_, Sep4_CYS39SNC_, and Sep6_CYS144SNC_ have an increased solvent‐accessible surface area and an increase in the hydrophilic area. Taken together, S‐nitrosylation changes the Rg values of target proteins and the accessible areas of the protein solvent, thus affecting the interaction between the target protein and the substrate. These changes could affect the accessible area of septins, thus affect the interaction between each other and consequently affect septin ring formation.

### 2.16 S‐nitrosylation Site C144 of Sep6 Protein is Important for Virulence

S‐nitrosoproteomic analysis and target validation confirmed that septin proteins are targets of S‐nitrosylation, then we selected Sep6 protein for functional analysis of the S‐nitrosylation sites. To validate the identified S‐nitrosylation modification site of Sep6 (C144), we mutated this site by changing C to S and transformed the mutated construct into the ∆*sep6* mutant. The biotin conversion assay showed reduced S‐nitrosylation modification in the S‐nitrosylation modification site mutant ∆*sep6/SEP6^C144S^* compared to the ∆*sep6/SEP6* strain (Figure 6M), suggesting that GSNOR may regulate de‐nitrosylation of Sep6 through recognition of the C144 site. The C144 S‐nitrosylation site of Sep6 protein plays an important role in the regulation of the growth and conidia formation of *M. oryzae* (Figure S10A–C, Supporting Information). Moreover, C144 of Sep6 was also required for pathogenicity and disease spot expansion (Figure 6N,O). Further analysis showed that the infection process of the mutant strain ∆*sep6/SEP6^C144S^* was slowed down (Figure S10D,E, Supporting Information). Importantly, C144S mutation also resulted in Sep6 distributing as some granules without forming a ring (Figure 6K,L). Taken together, these results showed that the S‐nitrosylation site C144 is a key site for the biological functions of Sep6.

### 2.17 GSNOR Inhibitor N6022 Effectively Suppresses the Infection of *M. oryzae*

**Figure 8:**
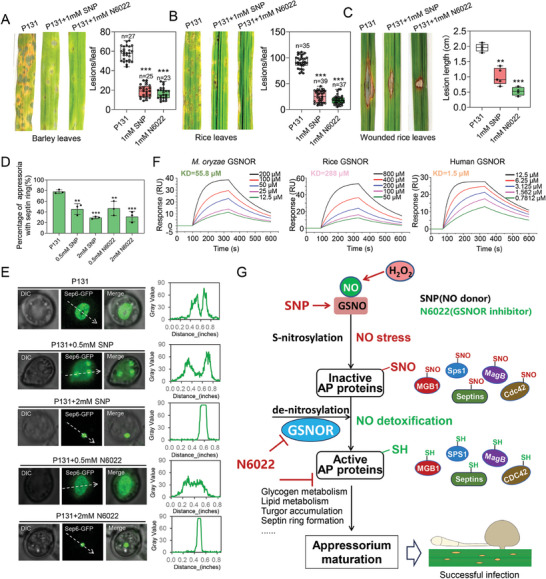
Effects of N6022 and SNP on infection structure and host pathogenicity. A) Lesions formed on barley leaves after treatment with 1 mm SNP or N6022. The lesions are observed and the number of lesions is counted after 5 days of inoculation. Asterisks indicate significant differences (*** *P* < 0.001). B) Lesions formed on rice leaves after treatment with 1 mm SNP or N6022. The lesions are observed and the number of lesions is counted after 5 days of inoculation. Asterisks indicate significant differences (*** *P* < 0.001). C) Virulence test on wounded rice leaves. Rice leaves were gently scraped with a needle and inoculated with spore solution treated with 1 mm SNP or 1 mm N6022. The length of lesions was measured and recorded after 4 days of inoculation. Asterisks indicate significant differences (** *P *< 0.01,*** *P* < 0.001). D) The percentage of septin‐ring formation was calculated for each treatment. Data presented are the mean ± standard errors from three biological replicates (*n* = 3), and significant differences compared with the WT are indicated by an asterisk (** *P* < 0.01, *** *P* < 0.001). E) Observation on the formation of appressorium septin ring after treatment with 0.5 and 2 mm SNP or N6022. Bar, 5 µm. F) SPR analysis of N6022 binding to GSNOR of *M. oryzae* and that of rice/human. G) A proposed model of de‐nitrosylation mediated appressorium formation in *M. oryzae*. During infection of *M. oryzae*, the appressorium formation accompanied with accumulation of massive NO. H_2_O_2_ also contributes to the accumulation of NO. NO and its bioactive donor S‐nitrosoglutathione (GSNO) modify appressorium (AP) proteins through S‐nitrosylation (‐SNO), leading them to inactive proteins. While this process is reversed by the S‐nitrosoglutathione reductase GSNOR‐mediated de‐nitrosylation process, which converts the modification site of ‐SNO into ‐SH form, and oxidized glutathione (GSSG), resulting an increased ratio of GSH/GSSG. The de‐nitrosylated AP proteins are activated for full function, which facilitates appressorium‐related cellular processes and appressorium maturation, leading to a successful infection.

N6022 is a novel drug with strong inhibitory activity against GSNOR, which is important for maintaining NO homeostasis. To provide ideas for the development of fungal inhibitors and disease control strategies based on NO signaling pathway, we tested the effects of N6022 and SNP on the pathogenicity of *M. oryzae*. Spray experiments on barley and rice by adding 1 mm N6022 or SNP into conidial solution of the wild‐type strain were performed. We found that the necrotic lesions on rice and barley leaves were significantly reduced when treated with 1 mm N6022 or 1 mm SNP (**Figure** **8**A,B). Inoculation of N6022 or SNP into conidial solution of the wild‐type strain onto the scratched rice leaves showed that N6022 or SNP‐treatment both severely reduced the lesion expansion, which was consistent with those of spray experiments (Figure 8C).

As the septin proteins can be regulated by S‐nitrosylation, we then investigated whether N6022 or SNP treatment could affect the subcellular localization of septins in appressorium. Our results showed that after treatment with different concentrations of N6022 and SNP, Sep6 failed to aggregate into a complete ring and both N6022 and SNP all reduced the frequency of septin ring formation of in appressorium by ≈40%−60% (Figure 8D,E). These results suggest that N6022 and SNP destabilized the formation of the septin ring. Overall, our findings suggest that N6022 and SNP disrupted the NO homeostasis in *M. oryzae*, thereby affecting appressorium‐related functions and ultimately blocking infection. To assess the selectivity of the GSNOR inhibitor N6022, we explored its interactions with GSNOR from various species. Employing surface plasmon resonance (SPR), we directly analyzed the binding between N6022 and the GSNOR protein. The findings revealed that N6022 binds to *M. oryzae* with a binding affinity of 55.8 µm. This interaction is less potent than that observed with human GSNOR (as a positive control with an affinity of 1.5 µm) but significantly stronger than its interaction with rice GSNOR (with an affinity of 288 µm), as depicted in Figure 8F.

## 3 Discussion

Although the functions and regulatory mechanisms of S‐nitrosylation in other species have been widely revealed, they were largely unknown in fungi. In this study, we found that in *M. oryzae*, intracellular NO equilibrium was essential for development and infection processes, especially the functional appressorium formation (Figures 1 and 3). The S‐nitrosoglutathione reductase GSNOR‐mediated de‐nitrosylation process played a key role in scavenging excessive intracellular NO accumulation or nitrosative stress. Based on iodoTMT switch labeling approach, we were able to identify a large number of S‐nitrosylated proteins in *M. oryzae*, some of which were reported key proteins for functional appressorium formation. We further found that GSNOR was required to activate these key appressorial proteins by removing S‐nitrosylation (Figure 5). Notably, proper protein structure and septin ring formation of the septin proteins were required by removing S‐nitrosylation (Figures 6 and 7). Our work provided the comprehensive understanding of S‐nitrosylation regulatory mechanism in the plant pathogenic fungi.

### 3.1 GSNOR‐Mediated de‐nitrosylation is Essential for NO Homeostasis and Appressorium Formation

We revealed the biological significance of NO‐mediated S‐nitrosylation, a new type of post‐translational modification, in the pathogenic process of *M. oryzae*. GSNOR is responsible for regulating the intracellular levels of S‐nitrosylated proteins and homeostasis of NO metabolism in animals and plants.^[^ ^8^, ^18^ ^]^ We found that *M. oryzae* GSNOR also mediates intracellular NO level through regulating the de‐nitrosylation process. GSNOR‐mediated de‐nitrosylation is important for asexual development, stress response, appressorium formation and invasive growth of *M. oryzae*. Currently, there are few reports on the roles of NO in plant pathogenic fungi, including in *M. oryzae* and *C. minitans*. For example, low dose of NO is found to be required in the germination and appressorium formation of *M. oryzae*,^[^ ^27^ ^]^ and it is also required for asexual spore formation of *C. minitans*.^[^ ^28^ ^]^ In this study, we found that high concentration of NO (addition of 5 mm SNP) is harmful for appressorium formation. Deletion of *GSNOR* led to a serious increase of NO level, which was also harmful for appressorium formation, but the NO scavenger cPTIO‐treatment can reverse this phenomenon (Figure 1A–E). Consistent with this, the addition of exogenous SNP and cPTIO respectively increased and decreased the protein S‐nitrosylation level in *M. oryzae*. Therefore, GSNOR is essential for keeping intracellular NO homeostasis for development and appressorium formation. Plants can employ nitrosative stress (NO burst) to suppress fungal infection.^[^ ^29^ ^]^ Coordinately, fungi also developed NO‐detoxification system to adapt to nitrosative stress, such as *M. oryzae* S‐(hydroxymethyl)‐glutathione dehydrogenase Sfa1 and flavohemoglobin MoFhb1 mediated NO detoxification^[^ ^15^, ^30^ ^]^ and *F. graminearum* transcription factor FgAreB.^[^ ^3a^ ^]^ However, the synthetic pathway of NO in *M. oryzae* remains elusive, this notion awaits proof by more genetic studies.

### 3.2 Proteomics Analysis Identified Infection‐Associated S‐nitrosylation Proteins

The main physiological role of NO is the protein S‐nitrosylation. However, less is known about the mechanism by which S‐nitrosylation regulates signaling in fungi. To date, more than thousands of S‐nitrosylated proteins have been identified by proteomics methods in mammal and plants.^[^ ^31^ ^]^ However, genome‐wide identification of S‐nitrosylated proteins has not been performed in fungi. We used an iodoTMT switch labeling approach to investigate the role of S‐nitrosylation modifications in *M. oryzae*. By using different samples from developmental stages and SNP treatment conditions, we were able to identify 483 S‐nitrosylated proteins with 741 S‐nitrosylation sites (Figure 4A). Interestingly, a large number of previously reported infection‐associated proteins were identified as S‐nitrosylated proteins, providing abundant information for future deciphering regulatory mechanism of S‐nitrosylation.

### 3.3 GSNOR‐Mediated de‐nitrosylation Regulates Functional Appressorium Formation

A number of proteins crucial for appressorium formation were identified as S‐nitrosylated proteins, including Pmk1‐MAPK signaling pathway upstream proteins (**Figure** **5**A). These appressorium‐associated proteins suggested a crucial role of S‐nitrosylation during appressorium stage. However, we propose that the regulation of S‐nitrosylation to these proteins may be negative. S‐nitrosylated AP proteins are normally inactive, during appressorium formation, they are activated by GSNOR‐mediated de‐nitrosylation. Then activated AP proteins regulate glycogen and lipid metabolism, turgor accumulation, septin ring formation to form functional appressorium (Figure 8E). Consistent with this, the Δ*gsnor* mutant showed defects of appressorium formation and maturation, as well as glycogen and lipid utilization, turgor accumulation, septin ring formation and appressorial adhesion.

We found that the S‐nitrosylation of septin proteins is a novel regulatory mechanism for the formation of septin ring, which is essential for penetration during infection of *M. oryzae*.^[^ ^26^, ^32^ ^]^ Our study proved four septin proteins (Sep3, Sep4, Sep5, and Sep6) as S‐nitrosylated proteins. GSNOR can interact with all of four septin proteins, and the septin ring formation was also affected in the ∆*gsnor* mutant, indicating that de‐nitrosylation of septin proteins positively regulates their function. S‐nitrosylation may block cysteine site and affecting formation of disulfide bonds, then affect intramolecular or intermolecular interactions intermolecular interactions of septin proteins, therefore affect septin ring construction. As a case study, we showed that the C144 S‐nitrosylation site of Sep6 protein was important for septin ring formation and infection of *M. oryzae*. PTM level regulation of septin proteins have been found to be important for assembly of septin ring in yeast and fungi.^[^ ^33^ ^]^ Core septins (ScCdc3, Cdc10, Cdc11 and Cdc12 and their orthologs) were well regulated by SUMOylation, acetylation, and phosphorylation.^[^ ^33b^ ^]^ Here, we found a novel PTM, S‐nitrosylation, also modified core septins, providing novel insight into the regulation of fungal septin ring assembly.

### 3.4 Disturbing of Appressorial NO Homeostasis Could be an Effective Disease Control Strategy

We propose that targeted breaking NO homeostasis by NO donors, NO scavengers, as well as chemical inhibitors of GSNOR, should be effective methods for fungal disease control. Studies have reported that NO was involved in pathogenicity in animal pathogens and controlled spore germination and infection structure development in plant pathogenic fungi.^[^ ^34^ ^]^ Our study also showed that the NO homeostasis is also essential for development and infection of *M. oryzae*. Pathogenicity tests showed that both NO donor SNP and GSNOR inhibitor N6022 can reduce the virulence of *M. oryzae*, although this process does not exclude the contribution of N6022 to plant resistance. Our results suggest that disrupting the NO homeostasis in *M. oryzae* has a negative impact on the growth and development of the pathogen and the formation of infection structures (septin ring) (Figures 1 and 8G). Therefore, the use of NO donors or chemical inhibitors of GSNOR are effective in rice blast control. Considering that harmful of excessive NO to different fungal pathogens could be similar, and that both septins and GSNOR are conserved in fungi, this strategy may be a broad‐spectrum strategy for controlling of fungal diseases. It is worth to mention that, the GSNOR inhibitor, such as N6022, is likely to be toxic to various organisms, including the plant host rice and barley. While on the other hand, the plant host could be more tolerant to N6022 than the fungus, when we treated rice or barley seedlings with 5 or 10 mm N6022 for one week, the growth of the plant was not affected (Figure S11, Supporting Information). Considering that 10 mm is much higher than that of 1 mm used for fungal control, it is possible for utilization. However, standard safety evaluation to human and environment should be tested in the future. It is also possible to develop more fungal‐specific inhibitors for GSNOR, for example, based on the structural differences or by gene silencing strategy based on distinct RNA sequences in different organisms.

## 4 Experimental Section

### Gene Knockout and Complementation

P131 was used as a wild‐type strain of *M. oryzae*. The *GSNOR* gene was deleted using a split‐PCR strategy.^[^ ^35^ ^]^ Information of all strains and vectors was shown in Tables S3 and S4 (Supporting Information). To test the stress response of the mutants, different strains were inoculated on CM plate supplanted with 0.1 mg ml^−1^ Calcofluor White [CFW], 0.2 mg ml^−1^ Congo Red [CR], 0.5 m NaCl, 1.0 m Sorbitol or different concentrations of H_2_O_2_. The colony diameter was measured after 5‐day post‐inoculation (dpi).

### Quantitative Real‐Time PCR Analysis

To detect the expression of *GSNOR* gene in *M. oryzae*, total RNA was extracted from samples collected from different developmental stages (mycelium, conidium, appressorium at 3 and 12 h, and invasive hyphae at 18, 24, and 48 hpi). Then the cDNA template was prepared for qRT‐PCR analysis using a SYBR Green PCR Master Mix Kit (Takara, Dalian, China) on an ABI 7500 real‐time PCR system (Applied Biosystems, Foster City, CA, USA).

### Staining Assays

The mycelia were stained with 10 µg ml^−1^ CFW (Sigma‐Aldrich, St. Louis, MO, USA) for 5 min and examined under a fluorescence microscope (Ni90, Nikon) to observe the cell length. For glycogen and lipids staining, conidial suspensions were dropped onto hydrophobic coverslip and incubated for 0, 2, 4, 6, 8, 12, 18, and 24 hpi in a dark moist chamber at 28 °C, followed by staining with KI/I_2_ solution (50 mg ml^−1^ KI, 5 mg ml^−1^ I_2_) or Nile red solution (2.5 µg ml^−1^ Nile red, pH 7.5). For NO staining, samples from different developmental stages of *M. oryzae* were stained using 5 µm DAF‐FM DA (Biyuntian, Beijing, China) dye and incubated at 37 °C for 20 min, followed by washing off the dye. Photographs were taken under a laser confocal microscope TCS SP8 (Leica Microsystems, Mannheim, Germany). For mitochondrial staining, spores and mycelial samples of GFP‐GSNOR strain were stained using MitoTracker Red CMXRos (Coolaber, China) dye, incubated at 37 °C for 20 min, and then washed to remove the dye. Images were captured using a laser confocal microscope TCS SP8 (Leica Microsystems, Mannheim, Germany).

### Yeast Two‐Hybrid Experiment

The full‐length cDNA of *SEP3*, *SEP4*, *SEP5*, and *SEP6* were amplified and connected to the yeast expression vector pGBKT7. The full‐length cDNA of *GSNOR* was amplified and connected with the yeast expression vector pGADT7. The AD and BD vectors were co‐transformed into yeast strain AH109. The bacterial solution packets were inverted on SD/‐Leu/‐Trp and SD/‐Leu/‐Trp/‐His (Clontech, San Francisco, CA, USA) dishes and cultured at 30 °C for 3 days to observe the growth of colonies. The grown single colonies were transferred to a new SD/‐Leu/‐Trp/‐His medium, stained with 5 µl X‐α‐gal, and cultured at 30 °C for 2 days in dark to observe the results. The interaction between pGBKT7‐53 and pGADT7‐T were used as a positive control, and the interaction between pGBKT7‐Lam and pGADT7‐T were used as a negative control.

### SNP, cPTIO, and N6022 Treatment

To observe the effects of SNP (Biyuntian, Beijing, China), cPTIO (Sigma‐Aldrich, St. Louis, MO, USA), and N6022 (MedChemExpress, USA) treatments, spore suspension (2 × 10^5^ spores ml^−1^) of different strains was added with each reagent, then dropped on the hydrophobic coverslip to calculate the appressorium formation rate at 24 hpi. To observe infectious hyphae, spore suspension (1 × 10^5^ spores ml^−1^) containing each reagent was dropped on the barley epidermis, and the expansion was observed at 28 hpi. For rice inoculations, spore suspension (5 × 10^4^ spores ml^−1^ in 0.025% Tween 20) containing each reagent was sprayed onto the leaves of one‐month‐old rice seedlings (*Oryza sativa* cultivar “LTH”). The rice plants were then incubated at 28 °C for 5 days under full humidity to evaluate disease level. For scratch experiment, rice seedlings were punctured for inoculating spore suspension (1 × 10^5^ spores ml^−1^ in 0.025% Tween 20) containing each reagent, and disease lesions were observed at 5 hpi at 28 °C in full humidity.

### Assay for GSH, GSSG, and NO

According to the manufacturer's instructions, a GSH and GSSG assay kit from Solarbio, China, was used to assess the amount of GSH in the cortex. Thermo Scientific Multiskan FC, OD = 593 nm, colorimetric microplate reader was used to measure the content. Total Glutathione‐GSSG×2 was used to calculate the GSH content of the test samples. GSH content was given as µg g^−1^. The NO content was determined using a NO content assay kit (Abbkine Scientific, Wuhan) based on the improved Griess method principle.

### Proteomics Analysis of S‐nitrosylation Proteins

Protein labelling was performed according to the manufacturer's protocol for Iodo‐TMT kit. Briefly, 1 mg of the protein extracts was blocked for 1 h at room temperature using a blocking reagent with 1% SDS and 50 mm iodoacetamide (IAA) and free thiols were blocked. The sample was precipitated with 3 volumes of cold acetone and washed three times. The S‐nitrosothiols in the protein were reduced to free thiols by the addition of the reducing reagent sodium ascorbate (20 mm, 37 °C) and the samples were subsequently labeled with IodoTMT (Thermo Fisher Scientific, Rockford, USA) for 1 h at room temperature and then precipitated with acetone. The protein pellet was dissolved in wash buffer, all these steps were performed in the dark. Biotinylated proteins were digested with trypsin (Promega, Madison, WI, United States) overnight at 37 °C then mixed with 30 µL of streptavidin beads (Thermo Fisher Scientific, Rockford, USA) and incubated overnight at 4 °C. Wash the beads four times with a washing buffer (neutralization buffer plus 600 mm NaCl). S‐nitrosylated proteins could be purified with streptavidin beads (anti‐TMT antibody resin), which bind biotin with very high affinity and specificity. The proteins were then eluted with 20 mL of elution buffer and followed by mass spectrometry analysis using an LTQ‐Orbitrap Velos mass spectrometer (Thermo Fisher Scientific, Waltham MA, USA) connected to an Easy‐nLCII (Thermo Fisher Scientific, Bremen, GA, USA).

### Biotin Labeling of S‐nitrosylated Proteins

S‐nitrosylated proteins were analyzed using a kit (Cayman, USA) based on the biotin‐ switch assay as described previously.^[^ ^36^ ^]^ Briefly, ≈100 to 200 uL of the protein extracts was blocked for 1 h at room temperature using a blocking reagent whose free thiols were blocked. The sample was precipitated with cold acetone and washed three times. The S‐nitrosothiols in the protein were reduced to free thiols by the addition of the reducing reagent sodium ascorbate and the samples were subsequently labeled with maleimide‐biotin for 1 h at room temperature and then precipitated with acetone. The protein pellet was dissolved in wash buffer, all these steps were performed under indirect light. The biotinylated proteins were then mixed with 30 µL of Streptavidin beads (ABclonal Technology) and incubated overnight at 4 °C. Wash the beads four times with a washing buffer. S‐nitrosylated proteins could be purified with streptavidin beads, which bind biotin with very high affinity and specificity. The protein was then eluted and western blotted with appropriate antibodies.

### Data Analysis

The MS raw files were processed and searched using MaxQuant version 1.5.2.8 (MPI in Biochemistry, Martinsried, Germany) software. Proteins were identified by querying against the full sequence of *M. oryzae* 70‐15 (http://fungi.ensembl.org/Magnaporthe_oryzae/Info/Index). The S‐nitrosylated proteins were classified by GO annotation (http://www.geneontology.org/). The KEGG database was used to annotate protein pathways (https://www.genome.jp/kegg/) and was mapped on the KEGG pathway database using KEGG MAPPER. The STRING database was used to generate protein interaction networks of S‐nitrosylated proteins which were visualized by Cytoscape software (version 3.4.0.).^[^ ^37^ ^]^

### Co‐IP Assay

The pKNRG‐*SEP3*, pKNRG‐*SEP4*, pKNRG‐*SEP5*, and pKNRG‐*SEP6* vectors controlled by their native promoters (Table S3, Supporting Information) were constructed and respectively co‐transformed with pKNFLAG‐*GSNOR* into P131. Correct transformants were incubated in liquid CM at 28 °C for 48 h at 150 rpm. Around 1 g of fresh mycelia was harvested and washed with sterile water. Total proteins were extracted using IP cell lysate (Biyuntian, Beijing, China) and cultured for 2 h at room temperature after the addition of 10 µl anti‐FLAG beads (Bimake, Beijing, China) to capture GFP:Sep3, GFP:Sep4, GFP:Sep5, and GFP:Sep6 protein complex. The isolated beads were washed three times in PBS‐T (phosphate buffered saline, Tween 20) buffer and three times in 50 mm TMAB (trimethylammonium bicarbonate, pH 8.5) buffer, followed by the addition of 100 µl of elution buffer to eluate immunoprecipitated proteins. SDS‐PAGE was then performed with appropriate antibodies.

### Immunoblotting Analysis

For protein extraction, ≈0.2 g of mycelium was frozen and ground to a powder in liquid nitrogen and resuspended in 1 ml of extraction buffer (10 mm Tris‐HCl [pH 7.5], 150 mm NaCl, 0.5 mm EDTA, 0.5% Triton X‐100) supplemented with 1 mm PMSF (Sigma‐Aldrich). Total protein was then separated on 10% SDS‐PAGE gel for western blotting. Primary antibodies used in this study include S‐nitrosylation detection‐HRP kit (Cayman Chemical, Michigan, USA), anti‐GFP antibody (1:5000, Sigma, St. Louis, MO, USA), anti‐FLAG antibody (1:5000, Sigma, St. Louis, MO, USA). The horseradish peroxidase‐conjugated goat anti‐mouse IgG (1:10 000, Abclonal, China) was used for a secondary antibody.

### Molecular Dynamics Simulations

All 3D protein structures used in the molecular dynamics simulations were obtained from the Alphafold2 model in the UniProt database (https://www.uniprot.org/). All simulations were based on the initial model and a total of seven systems were prepared for simulation using GROMACS (version 2022.5) in conjunction with the charmm36‐jul2022 all‐atom force field. The protein was then solvated in simple point charge (SPC) water molecules in a cubic box, with the box edges ≈1.0 nm from any atom of the protein, and additional Na^+^ and Cl^−^ ions were added to neutralize the charge of each system. Next, the simulations were performed under a constant temperature of 300 K, and the V‐rescale algorithm was used with a temperature coupling time constant of 0.1 ps. All bond lengths were constrained using the linear constraint solver (LINCS) algorithm. Measurement of Van der Waals interactions used a simple cut off at 1 nm, and long‐range electrostatic interactions were handled using the particle mesh Ewald (PME) method with a fourth‐order spline interpolation and a 0.16 nm Fourier grid spacing. Finally, each system was subjected to 50 ns of MD simulation, and the time step used in the simulations was 2 fs. Using GROMACS for protein radius of gyration and RMSD analysis, VMD (version 1.9.4a57) was used for protein structure visualization, and PyMOL (version 2.5.0) software was used for calculating protein solvent‐accessible surface area. The chemical structure of N6022 was retrieved from the PubChem Compound database (https://www.ncbi.nlm.nih.gov/pccompound/).

### Protein Purification

Recombinant Human GSNOR protein was purchased from Abcam Corporation (ab124573). The coding sequence of GSNOR was amplified from the cDNA of *M. oryzae* and rice and cloned into pET‐28a. For protein expression, the transformed *E. coli* BL21 (DE3) cell cultures at an optical density at 600 nm of 0.6 were treated with 1 mm isopropyl‐β‐D‐thiogalactopyranoside and incubated for 12 h at 16 °C. Purification was performed using the His Bind Kit (Biyuntian, Beijing, China) under non‐denaturing conditions, following the manufacturer's instructions.

### Surface Plasmon Resonance (SPR) Analysis

The interaction of the recombinant proteins with N6022 was detected by OpenSPRTM (Nicoya Lifesciences, Canada) at 25 °C. The chip was activated for 240 s with the mixture at a flow rate of 20 µL min^−1^. Dilute N6022 with the same analyte buffer buffer to 6 concentrations. N6022 was injected to sample channel at a flow rate of 20 µL min^−1^ for an association phase of 240 s, followed by 360 s dissociation. Repeat 6 cycles of analyte according to analyte concentrations in ascending order. After each cycle of interaction analysis, the sensor chip surface should be regenerated completely with 10 mm Glycine‐HCl as injection buffer at a flow rate of 150 µL min^−1^ for 10 s to remove the analyte, then next concentration cycle of the Analyte N6022 needs to repeat injection and Regeneration steps. The data were retrieved and analyzed with TraceDrawer.

### Statistical Analysis

Perform statistical analysis using Excel (Microsoft 2021) or Prism 9 (GraphPad) software. The number of biological replications and statistical methods were shown in the figure legend, and experiments were performed at least three times. Statistical significance was calculated using two‐sided unpaired Student's *t*‐test and ANOVA. All error bars represent the standard deviation (SD) of the mean. Asterisks in all graphs indicate statistical significance (* *p* < 0.05; ** *p* < 0.01; *** *p* < 0.001; ns, not significant).

## Conflict of Interest

The authors declare no conflict of interest.

## Author Contributions

H.H. and W.H. contributed equally to this work. X.‐L.C. conceived this study, designed the investigation, wrote the manuscript, and supervised the project. H.H. and W.H. conducted most of the experiments. Z.Q. assessed the molecular docking, X.D., Z.R., and M.Q. participated in part of the phenotypic analysis. H.L., L.Z., and J.H. participated in the design of the investigation.

## Supporting information

Supporting Information

Supplemental Table 1

Supplemental Table 2

Supplemental Table 3

Supplemental Table 4

## Data Availability

The data that support the findings of this study are available from the corresponding author upon reasonable request.

## References

[1] a) W. K. Alderton , C. E. Cooper , R. G. Knowles , Biochem. J. 2001, 357, 593; 11463332 10.1042/0264-6021:3570593 PMC1221991

[2] R. I. Astuti , R. Nasuno , H. Takagi , Adv. Microb. Physiol. 2018, 72, 29. 29778216 10.1016/bs.ampbs.2018.01.003

[3] a) Y. Jian , Z. Liu , H. Wang , Y. Chen , Y. Yin , Y. Zhao , Z. Ma , Nat. Commun. 2021, 12, 2576; 33958593 10.1038/s41467-021-22831-8 PMC8102577

[4] O. Lamotte , J. B. Bertoldo , A. Besson‐Bard , C. Rosnoblet , S. Aimé , S. Hichami , H. Terenzi , D. Wendehenne , Front. Chem. 2014, 2, 114. 25750911 10.3389/fchem.2014.00114 PMC4285867

[5] a) M. W. Foster , D. T. Hess , J. S. Stamler , Trends Mol. Med. 2009, 15, 391; 19726230 10.1016/j.molmed.2009.06.007 PMC3106339

[6] a) G. P. Ahern , V. A. Klyachko , M. B. Jackson , Trends Neurosci. 2002, 25, 510; 12220879 10.1016/s0166-2236(02)02254-3

[7] a) D. T. Hess , A. Matsumoto , S. O. Kim , H. E. Marshall , J. S. Stamler , Nat. Rev. Mol. Cell Biol. 2005, 6, 150; 15688001 10.1038/nrm1569

[8] a) E. Kwon , A. Feechan , B. W. Yun , B. H. Hwang , J. A. Pallas , J. G. Kang , G. J. Loake , Planta 2012, 236, 887; 22767201 10.1007/s00425-012-1697-8

[9] a) S. D. Barnett , I. L. O. Buxton , Crit. Rev. Biochem. Mol. Biol. 2017, 52, 340; 28393572 10.1080/10409238.2017.1304353 PMC5597050

[10] a) M. Benhar , Curr. Med. Chem. 2016, 23, 2602; 27356534 10.2174/0929867323666160627114839

[11] a) C. Lindermayr , S. Sell , B. Müller , D. Leister , J. Durner , Plant Cell 2010, 22, 2894; 20716698 10.1105/tpc.109.066464 PMC2947166

[12] E. Aranda , C. López‐Pedrera , J. R. De La Haba‐Rodriguez , A. Rodriguez‐Ariza , Curr. Mol. Med. 2012, 12, 50. 22082481 10.2174/156652412798376099

[13] J. Hu , X. Huang , L. Chen , X. Sun , C. Lu , L. Zhang , Y. Wang , J. Zuo , Plant Physiol. 2015, 167, 1731. 25699590 10.1104/pp.15.00026 PMC4378176

[14] R. A. Wilson , N. J. Talbot , Nat. Rev. Microbiol. 2009, 7, 185. 19219052 10.1038/nrmicro2032

[15] Z. Zhang , J. Wang , R. Chai , H. Qiu , H. Jiang , X. Mao , Y. Wang , F. Liu , G. Sun , PLoS One 2015, 10, 0120627. 10.1371/journal.pone.0120627 PMC4368689 25793615

[16] D. P. Arora , S. Hossain , Y. Xu , E. M. Boon , Biochemistry 2015, 54, 3717. 25996573 10.1021/bi501476n

[17] B. J. Cao , M. E. Reith , Eur. J. Pharmacol. 2002, 448, 27. 12126967 10.1016/s0014-2999(02)01908-8

[18] a) A. Feechan , E. Kwon , B. W. Yun , Y. Wang , J. A. Pallas , G. J. Loake , Proc. Natl. Acad. Sci. USA 2005, 102, 8054; 15911759 10.1073/pnas.0501456102 PMC1142375

[19] C. M. Thompson , R. Ceder , R. C. Grafström , Toxicol. Lett. 2010, 193, 1. 19963048 10.1016/j.toxlet.2009.11.023

[20] Y. Ding , D. M. Gardiner , D. Xiao , K. Kazan , Proc. Natl. Acad. Sci. USA 2020, 117, 11147. 32376629 10.1073/pnas.1918977117 PMC7245131

[21] J. S. Stamler , D. I. Simon , J. A. Osborne , M. E. Mullins , O. Jaraki , T. Michel , D. J. Singel , J. Loscalzo , Proc. Natl. Acad. Sci. USA 1992, 89, 444. 1346070 10.1073/pnas.89.1.444 PMC48254

[22] a) J. Astier , A. Kulik , E. Koen , A. Besson‐Bard , S. Bourque , S. Jeandroz , O. Lamotte , D. Wendehenne , Free Radical Biol. Med. 2012, 53, 1101; 22750205 10.1016/j.freeradbiomed.2012.06.032

[23] a) M. He , J. Su , Y. Xu , J. Chen , M. Chern , M. Lei , T. Qi , Z. Wang , L. S. Ryder , B. Tang , M. Osés‐Ruiz , K. Zhu , Y. Cao , X. Yan , I. Eisermann , Y. Luo , W. Li , J. Wang , J. Yin , S. M. Lam , G. Peng , X. Sun , X. Zhu , B. Ma , J. Wang , J. Liu , H. Qing , L. Song , L. Wang , Q. Hou , et al., Nat. Microbiol. 2020, 5, 1565; 32958858 10.1038/s41564-020-00790-y

[24] a) S. N. Rao , M. S. Head , A. Kulkarni , J. M. LaLonde , J. Chem. Inf. Model. 2007, 47, 2159; 17985863 10.1021/ci6004299

[25] M. Lobanov , N. S. Bogatyreva , O. V. Galzitskaia , Molekuliarnaia biologiia 2008, 42, 701. 18856071

[26] Y. F. Dagdas , K. Yoshino , G. Dagdas , L. S. Ryder , E. Bielska , G. Steinberg , N. J. Talbot , Science 2012, 336, 1590. 22723425 10.1126/science.1222934

[27] M. Samalova , J. Johnson , M. Illes , S. Kelly , M. Fricker , S. Gurr , New Phytol. 2013, 197, 207. 23072575 10.1111/j.1469-8137.2012.04368.x

[28] X. Gong , Y. Fu , D. Jiang , G. Li , X. Yi , Y. Peng , Fungal Genet. Biol. 2007, 44, 1368. 17897846 10.1016/j.fgb.2007.07.007

[29] a) M. Delledonne , Y. Xia , R. A. Dixon , C. Lamb , Nature 1998, 394, 585; 9707120 10.1038/29087

[30] Z. Zhang , Z. Hao , R. Chai , H. Qiu , J. Wang , Y. Wang , G. Sun , FEMS Microbiol. Lett. 2022, 369, fnab162. 35259230 10.1093/femsle/fnab162

[31] T. Y. Lee , Y. J. Chen , C. T. Lu , W. C. Ching , Y. C. Teng , H. D. Huang , Y. J. Chen , Bioinformatics 2012, 28, 2293. 22782549 10.1093/bioinformatics/bts436

[32] Y. K. Gupta , Y. F. Dagdas , A. L. Martinez‐Rocha , M. J. Kershaw , G. R. Littlejohn , L. S. Ryder , J. Sklenar , F. Menke , N. J. Talbot , Plant Cell 2015, 27, 3277. 26566920 10.1105/tpc.15.00552 PMC4682301

[33] a) C. Liu , Z. Li , J. Xing , J. Yang , Z. Wang , H. Zhang , D. Chen , Y. L. Peng , X. L. Chen , New Phytol. 2018, 219, 1031; 29663402 10.1111/nph.15141

[34] a) J. Wang , V. J. Higgins , Fungal Genet. Biol. 2005, 42, 284; 15749048 10.1016/j.fgb.2004.12.006

[35] R. S. Goswami , Methods Mol. Biol. 2012, 835, 255. 22183659 10.1007/978-1-61779-501-5_16

[36] a) J. Feng , C. Wang , Q. Chen , H. Chen , B. Ren , X. Li , J. Zuo , Nat. Commun. 2013, 4, 1529; 23443557 10.1038/ncomms2541

[37] P. Shannon , A. Markiel , O. Ozier , N. S. Baliga , J. T. Wang , D. Ramage , N. Amin , B. Schwikowski , T. Ideker , Genome Res. 2003, 13, 2498. 14597658 10.1101/gr.1239303 PMC403769

[38] Y. Perez‐Riverol , A. Csordas , J. Bai , M. Bernal‐Llinares , S. Hewapathirana , D. J. Kundu , A. Inuganti , J. Griss , G. Mayer , M. Eisenacher , E. Pérez , J. Uszkoreit , J. Pfeuffer , T. Sachsenberg , S. Yilmaz , S. Tiwary , J. Cox , E. Audain , M. Walzer , A. F. Jarnuczak , T. Ternent , A. Brazma , J. A. Vizcaíno , Nucleic Acids Res. 2019, 47, D442. 30395289 10.1093/nar/gky1106 PMC6323896

