# Supplementary material for: De‐nitrosylation Coordinates Appressorium Function for Infection of the Rice Blast Fungus
Source: Adv Sci (Weinh). 2024 May 5;11(26):2403894. doi: 10.1002/advs.202403894 (PMC11234416; doi:10.1002/advs.202403894)
Supplement: Supplementary file 1 — Supporting Information [file ADVS-11-2403894-s003.pdf]

ADVANCED SCIENCE Open Access

Supporting Information

for *Adv. Sci.*, DOI 10.1002/advs.202403894

De-nitrosylation Coordinates Appressorium Function for Infection of the Rice Blast Fungus

*Hong Hu, Wenhui He, Zhiguang Qu, Xiang Dong, Zhiyong Ren, Mengyuan Qin, Hao Liu, Lu Zheng, Junbin Huang and Xiao-Lin Chen**

Supplementary data: Hu et al. (2024). De-nitrosylation coordinates appressorium function for infection of the rice blast fungus

This Supplemental data includes:
Supplemental Figure S1 to S11
Supplemental Table S1 to S4

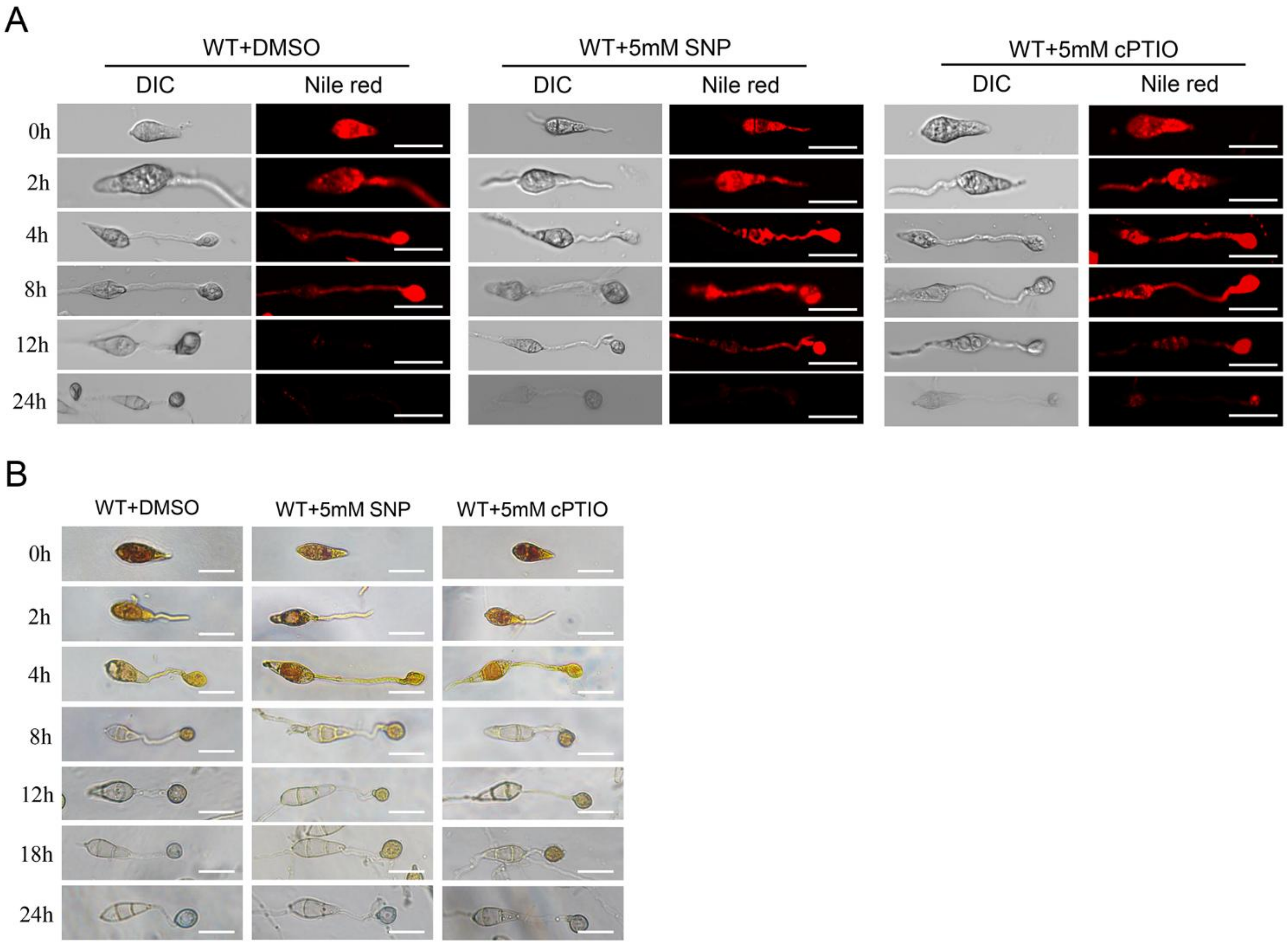

Figure S1. NO affects lipid droplet and glycogen utilization during appressorium maturation. A) Observation of lipid utilization during appressoria formation upon 5 mM SNP or 5 mM cPTIO treatment. Conidia and appressoria were stained with Nile Red and photographed at different time points. Bar, 20 µm. B) Observation of glycogen utilization during appressoria formation upon 5 mM SNP or 5 mM cPTIO treatment. The spores or appressoria were stained with I_2/KI and photographed at different time points. Bar, 20 µm.

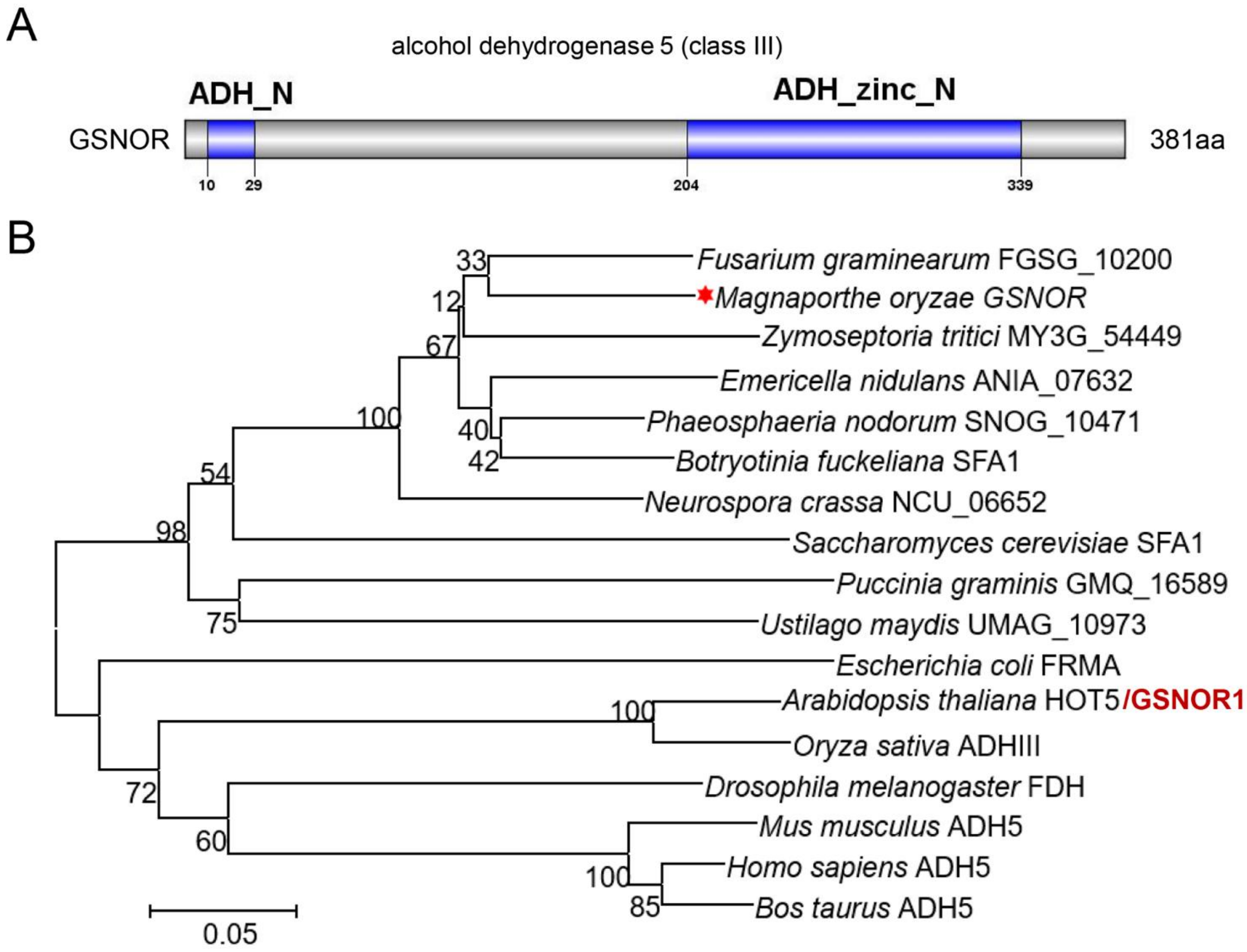

Figure S2. Analyses of GSNOR functional domain and systematic evolution. A) Domain analysis of *M. oryzae* GSNOR protein was performed using a simple modular architecture research tool (SMART). B) Phylogenetic analysis of *M. oryzae* GSNOR with homolog from other species was performed using molecular evolutionary genetics analysis v.5 (MEGA5). Bootstrap analyses with 1000 iterations were performed, and support for each node is shown. The numbers at the nodes represent the bootstrap values from the neighbor joining analysis.

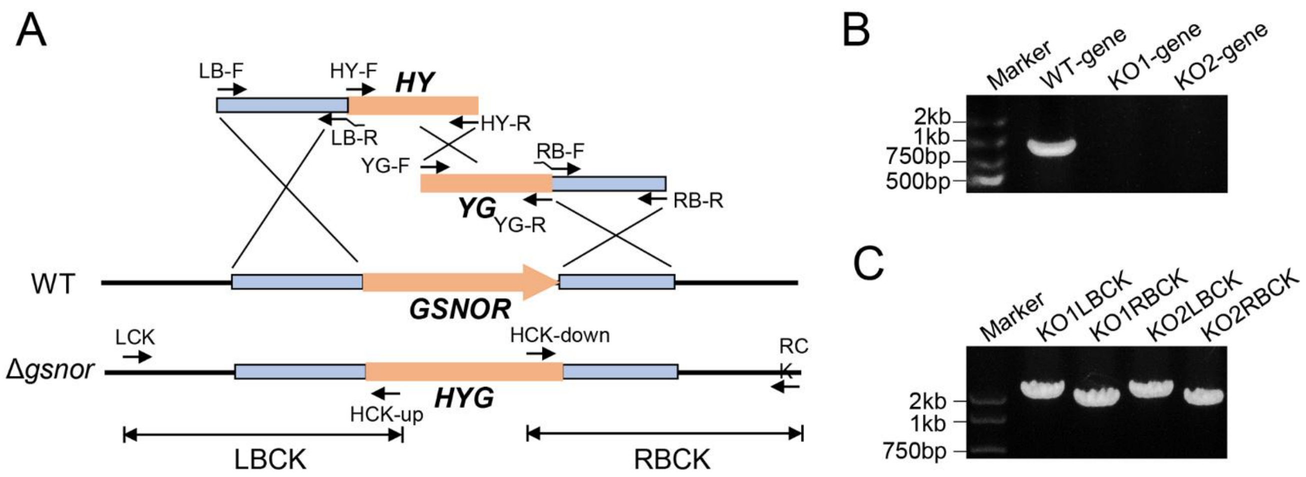

Figure S3. Replacement strategy and verification of GSNOR deletion mutants. A) A split-marker approach of gene replacement for *Magnaporthe oryzae GSNOR*. The white bars show the upstream and downstream genomic areas of the *GSNOR* coding sequence. The *GSNOR* coding sequence was amplified and fused to a piece of the hygromycin phosphotransferase (*HYG*) cassette. B) The *GSNOR* fragment of transformants and wild-type strain (WT) was amplified by RT-PCR. c PCR verification of flanking sequences in addition to the replacement fragment using LCK/HCK-up and RCK/HCK-down primer pairs.

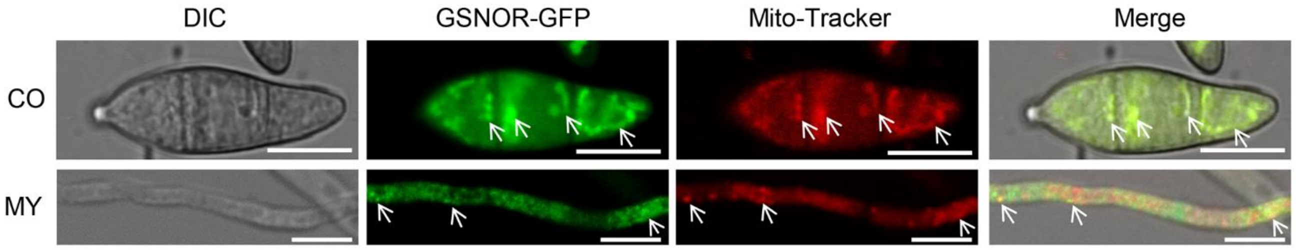

Figure S4 Subcellular localization of GSNOR. Observation of colocalization of GSNOR and mitochondrial marker mitotracker in mycelia and spore. Bar, 10 μm.

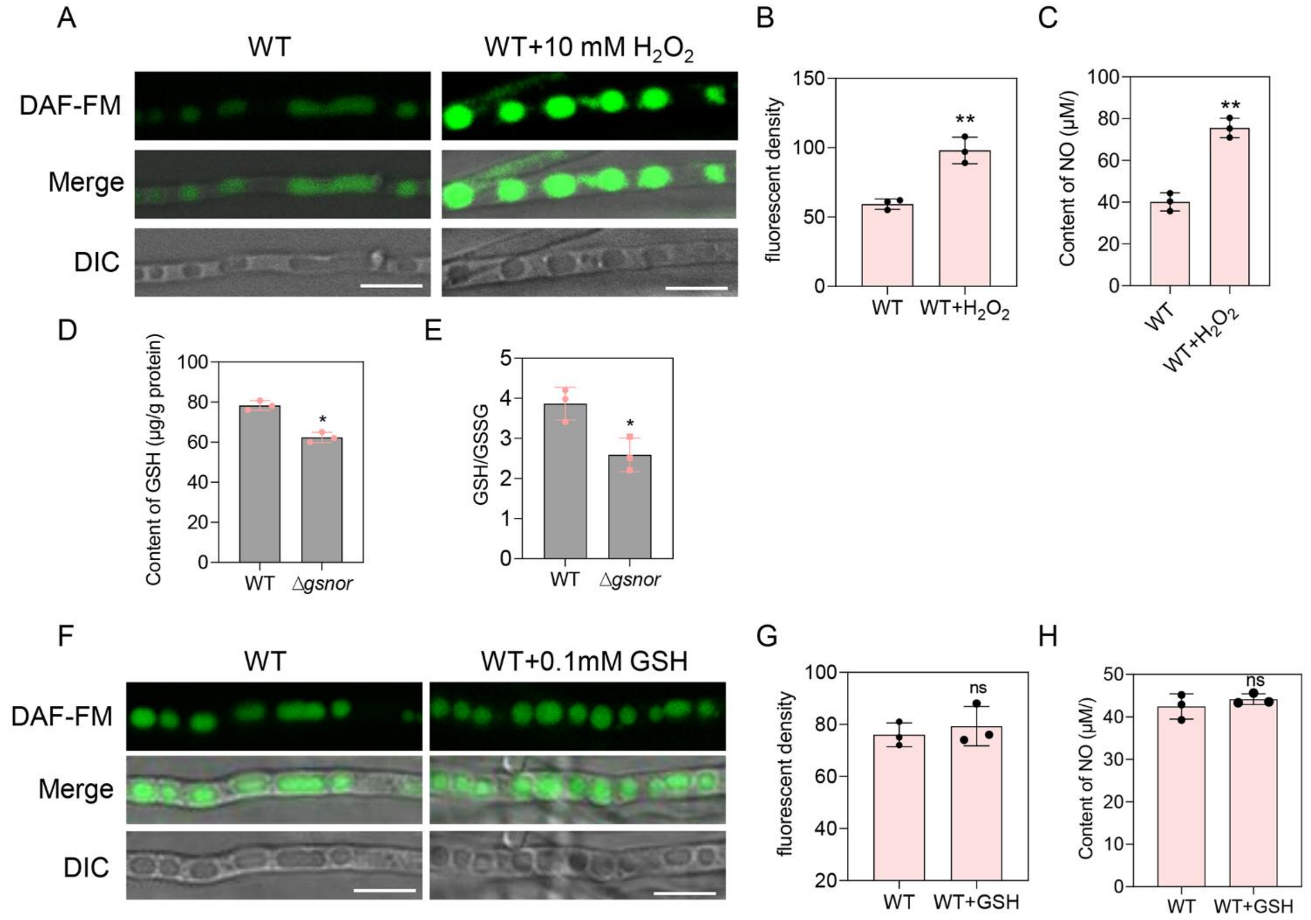

Figure S5. H_2O_2 stimulates the explosion of NO. A) Observation of NO content in wild-type (WT) and Δ*gsnor* during hyphae after 10mM H_2O_2 treatment. Bar, 10 μm. B) The bar chart shows the fluorescence intensity of DA-FM DA staining. Samples of each strain were measured using the ImageJ software. Data presented are the mean ± standard errors from three biological replicates (n = 3), and significant differences compared with the WT are indicated by an asterisk (**, $P < 0.01$). C) The concentration of NO in the mycelia after H_2O_2 treatment was determined by the improved Griess method. Data presented are the mean ± standard errors from three biological replicates (n = 3), and significant differences compared with no treatment are indicated by an asterisk (**, $P < 0.01$). D) Determination of GSH content in wild-type (WT) and Δ*gsnor*. E) Determination of GSH/GSSG ratio in wild-type (WT) and Δ*gsnor*.

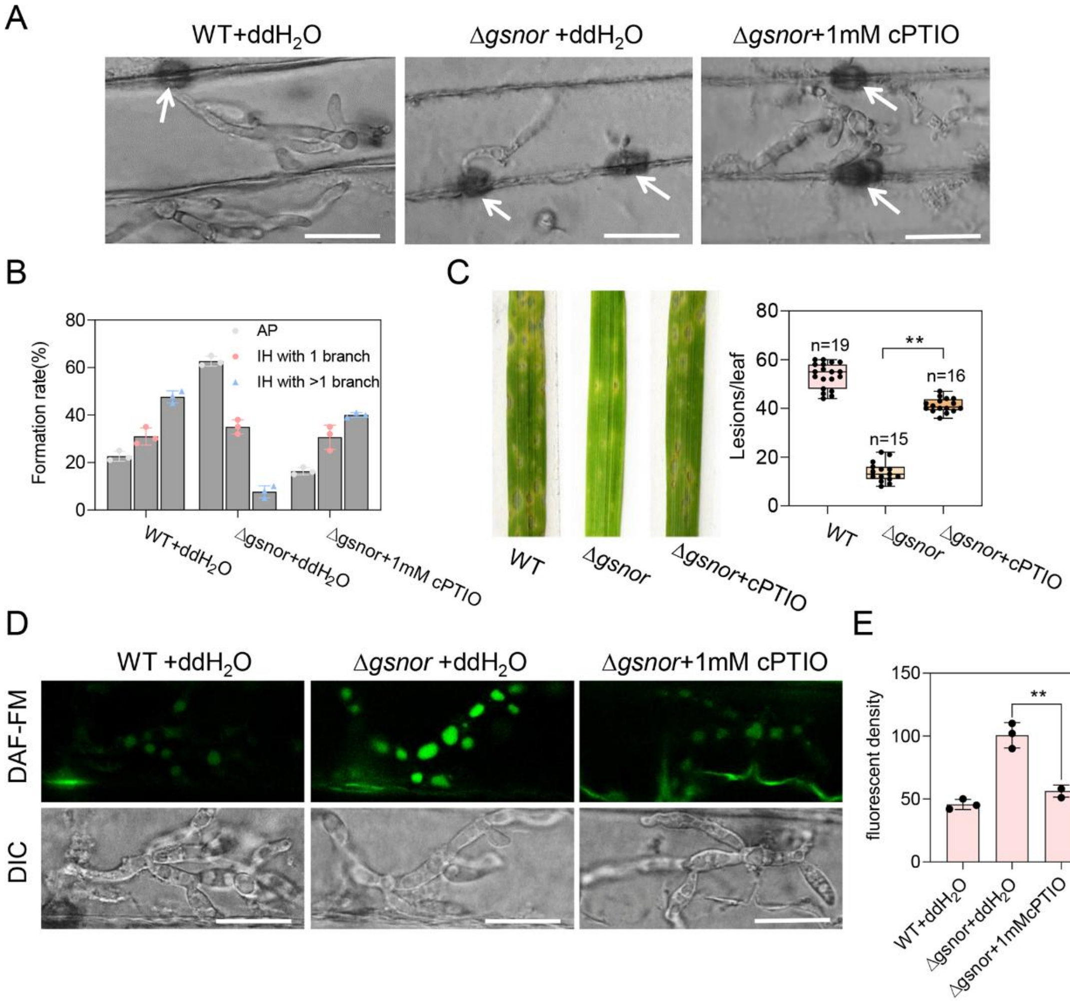

Figure S6. cPTIO can partially rescue the defects of Δ*gsnor.* A) Spores treated with 1mM cPTIO were inoculated with barley, and the invasive hyphae of wild-type (WT) and Δ*gsnor* were observed at 24h. Bar, 15 µm. B) Statistical data of different structures during the invasive hyphae were observed. AP, appressoria; IH, invasive hyphae. Data presented are the mean ± standard errors from three biological replicates (n = 3). C) Spores treated with 1mM cPTIO were inoculated with barley. The lesions are observed and the number of lesions is counted after 5 days of inoculation. Asterisks indicate significant differences (**, $P < 0.01$). D) Observation of NO levels in wild-type (WT) and Δ*gsnor* during invasive hyphae after cPTIO treatment. Bar,15 µm. F) The bar chart shows the fluorescence intensity of DA-FM DA staining. Samples of each strain were measured using the ImageJ software. Asterisks indicate significant differences (**, $P < 0.01$).

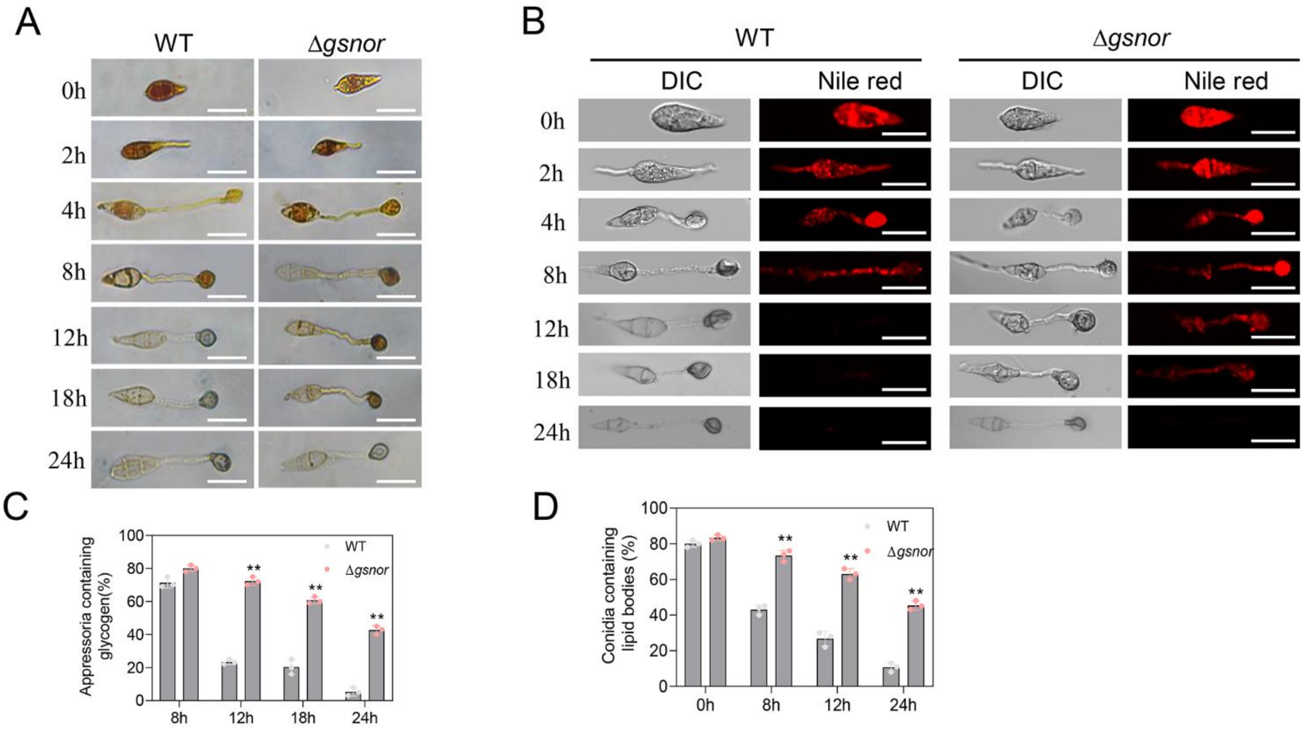

Figure S7. Deletion of *GSNOR* affects the utilization of glycogen and lipid. A) Observation of glycogen utilization during appressorium formation. The spores or appressorium were stained with I_2/KI and photographed at different time points. Bar, 20μm. B) Observation of lipid utilisation during appressorium formation. The spores or appressorium were stained with Nile Red and photographed at different time points. Bar, 20μm. C-D) Statistics showing the percentage of glycogen or lipids in conidia or appressorium during appressorium development of wild-type (WT) and Δ*gsnor*. Data presented are the mean ± standard errors from three biological replicates (n = 3), and significant differences compared with the WT are indicated by an asterisk (**, $P < 0.01$).

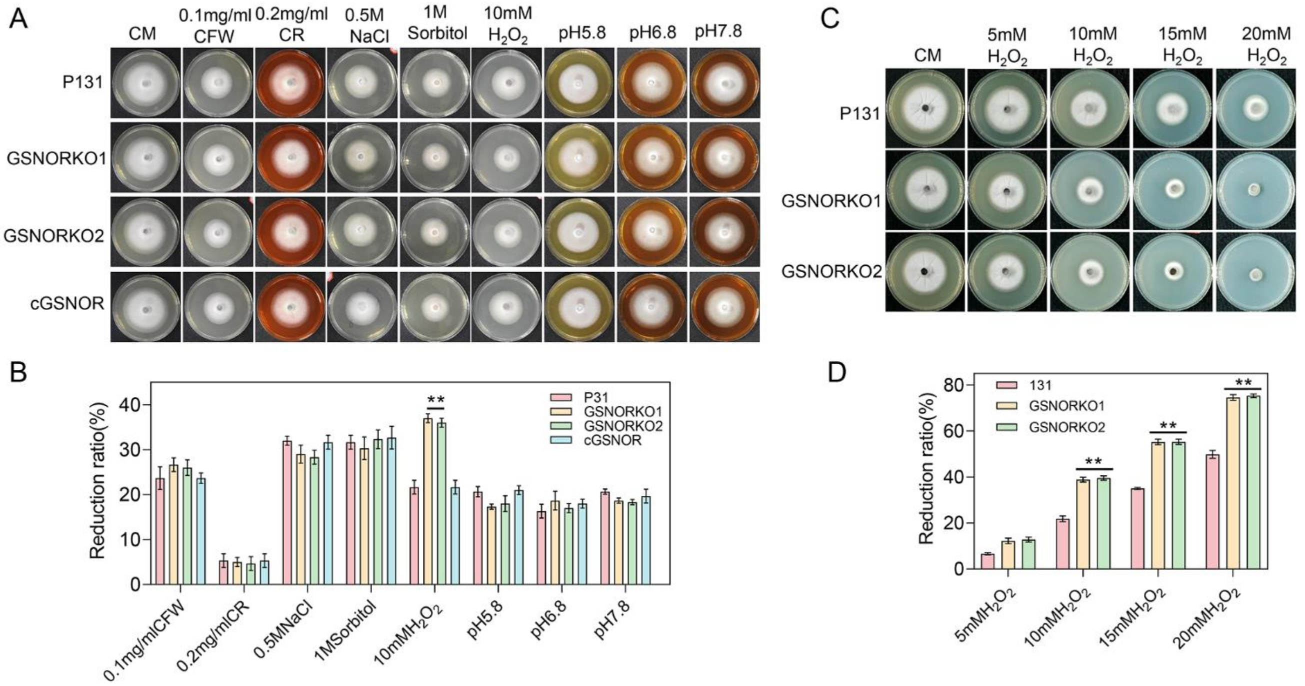

Figure S8. GSNOR is hypersensitive to hydrogen peroxide. A) Growth morphology of different strains on complete medium (CM) plates with different stress reagents or pH buffers. Observation was made 5 days after inoculation of hyphal agar plugs. B) Statistics of growth reduction rates of different strains on various stress reagents. For each strain, the reduction rate was calculated by comparing the colony diameter on treatments with that of CM without treatment. The means and standard errors were calculated by three independent repetitions. Asterisks indicate significant difference (**, $P < 0.01$). C) Growth morphology of different strains on complete medium (CM) plates with different concentrations of hydrogen peroxide. Observation was made 5 days after inoculation of mycelium. D) The growth reduction rates of different strains to different concentration of hydrogen peroxide was measured. For each strain, the reduction rate was calculated by comparing colony diameters of treated and untreated CM. The means and standard errors were calculated by three independent repetitions. Asterisks indicate significant difference (**, $P < 0.01$).

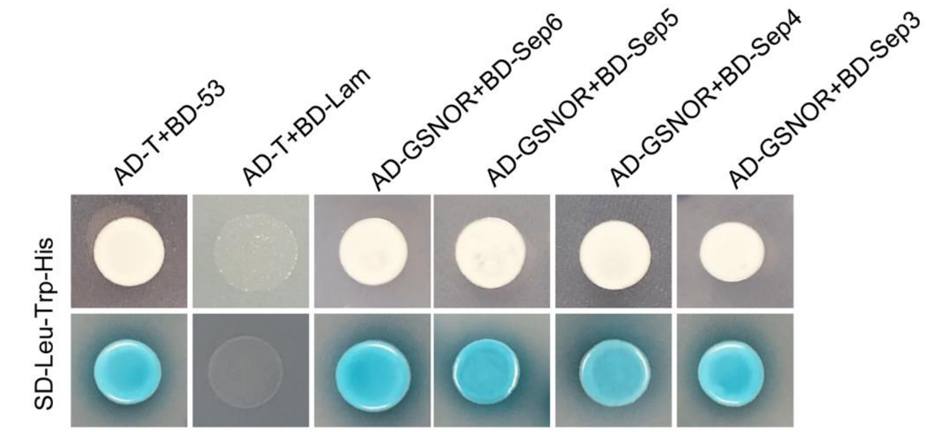

Figure S9 Interactions between GSNOR and septins by yeast two hybrid assay. The interaction of GSNOR with Sep3, Sep4, Sep5 and Sep6 was detected by yeast two-hybrid assay. The blue interaction between pGBKT7-53 (BD-53) and pGADT7-T(AD-T) was used as the positive control, and interaction between pGBKT7-Lam (BD-Lam) and pGADT7-T was used as the negative control. Positive colonies on SD/−Leu−Trp−His were stained with X-α-gal.

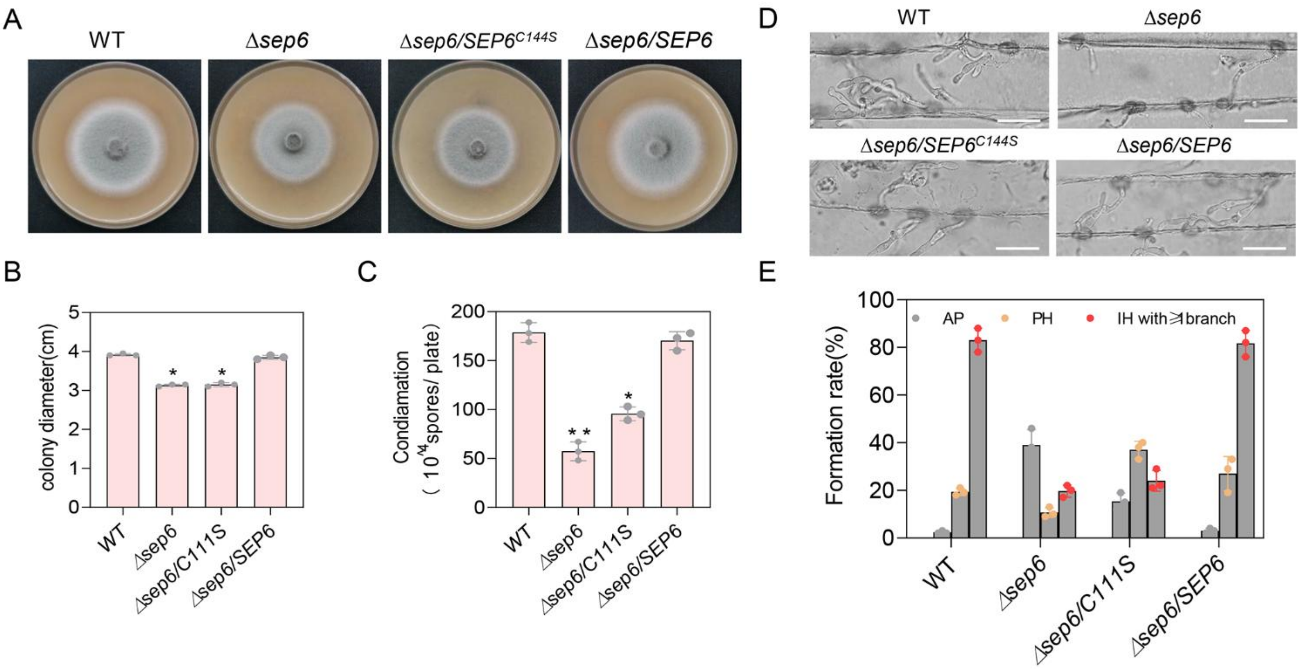

Figure S10. The C144S site mutation in septin-associated protein (Sep6) affects the pathogenic process of *M. oryzae*. A) Colony morphology of the Δ*sep6/SEP6*^{C144S} strain. The different strains were cultured on oatmeal tomato agar (OTA) medium at 28°C for 5 d. B) Colony diameter statistics of different strains. Data presented are the mean ± standard errors from three biological replicates (n = 3). Asterisks indicate significant difference (*, $P < 0.05$). C) Statistics of conidiation capacity of different strains. Conidia were collected from strains grown on OTA plates. Means and standard errors were calculated from three independent experiments. Significant differences compared to the wild type are indicated with an asterisk (*, $P < 0.05$; **, $P < 0.01$). D) Infection process of different strains infecting barley epidermal cells. E) The percentage of appressorium (AP), primary hyphae (PH) and invasive hyphae (IH) formation in different strains within 24 hours. Data presented are the mean ± standard errors from three biological replicates (n = 3).

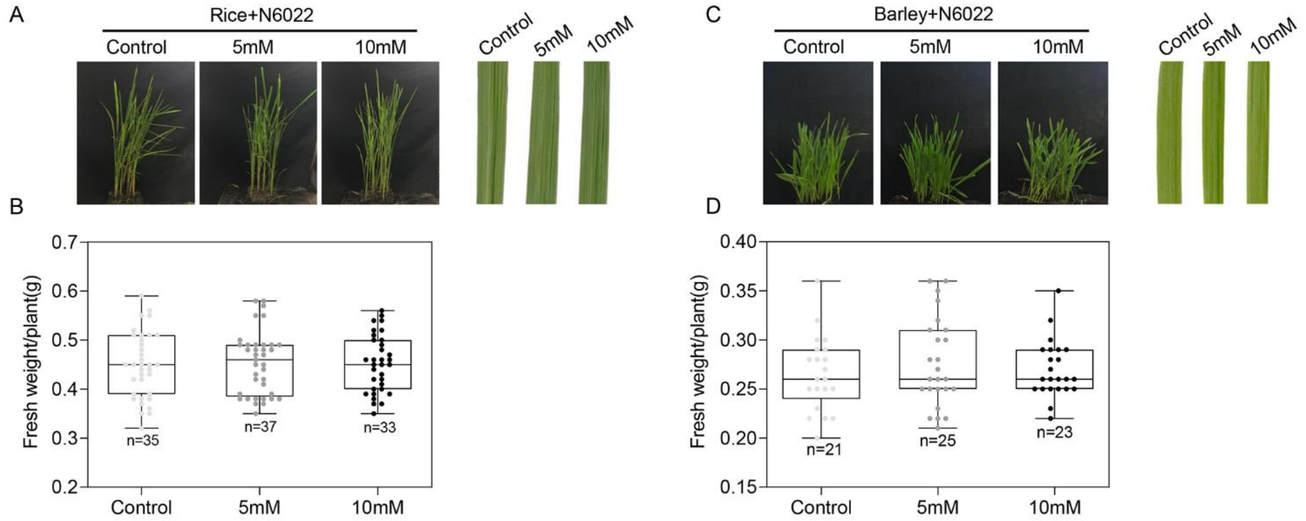

Figure S11. Phytotoxicity assays of GSNOR inhibitors N6022 in rice and barley. A) Seedlings of rice after seven days of spray with 5mM or 10mM N6022. B) Fresh weight per plant of rice seedlings exposed to 5 mM or 10 mM N6022 at 7 dpi. Means and standard deviations were calculated from the number of independent replicates as indicated. Data were analyzed with Student's t-test. C) Seedlings of barley after five days of spray with 5 mM or 10 mM N6022. D) Fresh weight per plant of barley seedlings exposed to 5 mM or 10mM N6022 at 5 dpi. Means and standard deviations were calculated from the number of independent replicates as indicated. Data were analyzed with Student's t-test.

Supplementary Tables

Table S1 List of proteins increased in nitrosylation level in the Δ*gsnor* mutant.
Table S2 S-nitrosylated protiens involved in pathogenesis of *M. oryzae*.
Table S3 All strains used in this study.
Table S4 Plasmids used in this study.
